# OzNOxESI: A Novel Mass Spectrometry Ion Chemistry for Elucidating Lipid Double-Bond Regioisomerism in Complex Mixtures

**DOI:** 10.1021/acs.analchem.4c05940

**Published:** 2025-01-16

**Authors:** Ryan A. Smith, Ashraf M. Omar, Fayaj A. Mulani, Qibin Zhang

**Affiliations:** † Center for Translational Biomedical Research, University of North Carolina at Greensboro, Kannapolis, North Carolina 28081, United States; § Department of Chemistry & Biochemistry, University of North Carolina at Greensboro, Greensboro, North Carolina 27402, United States

## Abstract

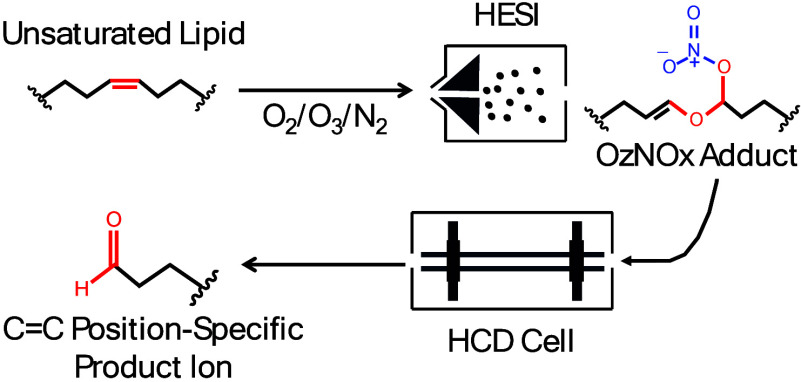

Double bond (C=C) position isomerism in unsaturated lipids can indicate abnormal lipid metabolism and pathological conditions. Novel chemical derivatization and mass spectrometry-based techniques are under continuing development to provide more accurate elucidation of lipid structure in finer structural detail. Here, we introduce a new ion chemistry for annotating lipid C=C positions, which is highly efficient for liquid chromatography–mass spectrometry-based lipidomics. This ion chemistry relies on the online derivatization of lipid C=C with ozone and nitrogen oxides upon fragmentation by tandem mass spectrometry, yielding characteristic product ions capable of unambiguously annotating C=C regioisomers. The new workflow was thoroughly evaluated with various glycerophospholipids and fatty acids and applied to human plasma lipid extract, successfully identified and quantified 270 glycerophospholipid and 36 fatty acid C=C isomers with an in-house developed software, OzNOx Companion, for automated data analysis.

## Introduction

Lipids play key roles in biological systems including cell membrane functionality, energy storage, and cell signaling. They are very structurally diverse, with a common theme that most are amphiphilic molecules possessing a hydrophilic headgroup linked to one or more hydrophobic tail(s).^1^ The diversity of lipids comes from different head groups (lipid classes), different tails (e.g., fatty acyls, ether-linked alkyl chains, branched alkyl chains, and sphingoid bases), and various forms of isomerism, including structural isomerism, regioisomerism, and stereoisomerism.^1^ Such structural complexity is reflected in differences in membrane composition, tissue and organism specificity, and the diversity of lipid functions and metabolism. At the double-bond (C=C) position level, differential expression of the fatty acyls in phosphatidylcholines (PC) was observed with FA 18:1(*n*-7) more expressed in cancerous than in normal mouse breast tissue;^2^ unusual lipid metabolism was observed in different cancer cells in that the nonmethylene interrupted fatty acid FA 20:2(*n*-7,12) is only found in LNCaP cells, but not in MCF7 cells;^3^ a profiling of total fatty acids in human plasma revealed that the relative abundance of FA 16:1(*n*-10) and FA 18:1(*n*-10) was elevated in patients with type 2 diabetes compared to healthy individuals.^4^ These changes could be easily masked if the lipid structure is not resolved at the C=C level.

To truly comprehend and monitor biological lipidome variation, it is necessary to develop analytical methods that can tackle all levels of structural variation. Liquid chromatography paired with tandem mass spectrometry (LC-MS^2^), particularly high resolution mass spectrometry, largely differentiates lipid class and tail content,^5−8^ with partial resolution of structural isomerism for some lipids.^9,10^ C=C regioisomerism has been a target for intensive method developments in recent years, including direct analysis methods with specialized mass spectrometers such as ultraviolet photodissociation,^11,12^ radical induced dissociation using atomic oxygen, hydroxyl radicals, and hydrogen radicals;^13^ and in-solution chemical derivatization methods such as epoxidation,^14,15^ aziridination,^16−19^ and Paternò–Büchi reaction^20−23^ followed by tandem mass spectrometry of the derivatized lipids. In particular, the latter can also be performed online inside a mass spectrometer. For its simplicity in data interpretation, online ozonolysis has steadily gained attention. This technique was initially developed with ozone in the ESI nebulizing gas, a technique known as OzESI.^24,25^ This approach generates product ions that indicate lipid C=C position but are often ambiguous with respect to their precursor compound if multiple unsaturated lipids are ionized together by either direct infusion or coelution of lipids in LC-based separation of complex lipid samples. Mass selection of lipids prior to ozonolysis by introducing ozone to the collision cell, also known as ozone-induced dissociation (OzID), largely solved the ambiguity issue in assigning C=C specific product ions to their precursor lipids.^26^ However, the low pressure of ozone inside the mtorr high vacuum collision cell renders this first order ozonolysis reaction low efficiency in production of C=C characteristic ions and challenging to couple with LC separation. Improvements to reaction efficiency include using traveling wave enabled devices by either accumulating ions in a stacked ring ion guide-equipped collision cell to increase the reaction time between ozone and lipid ions,^27^ or using the ion mobility region as a high pressure (sub torr) ozone reactor for efficient coupling with LC.^28^ In addition, online ozonolysis inside a high pressure ion funnel followed by ion mobility separation prior to tandem mass spectrometry was also implemented,^29^ which has additional benefit of further precursor ion separation using ion mobility but requires specialized IMS mass spectrometers.

To address the ambiguity issues in C=C isomer assignment in LC-OzESI-MS, here in this work we present OzNOxESI, a novel ion chemistry occurring in the ionization source exclusively in the presence of ozone, nitrogen, and oxygen using a simple OzESI setup, for unambiguous identification of lipid C=C position at the LC time scale. The OzNOx adduct formed with unsaturated lipids generates C=C position-specific product ions when the mass is selected for fragmentation in a mass spectrometer. In combination with OzNOx Companion, a Python tool for automated processing of the LC-OzNOx-MS^2^ data, we demonstrate the ability of this novel workflow in confident identification and quantification of glycerophospholipid (GPL) and fatty acid C=C position isomers in complex lipid standards and human plasma lipid extract.

## Results

### Novel OzNOxESI Ion Chemistry

**Figure 1:**
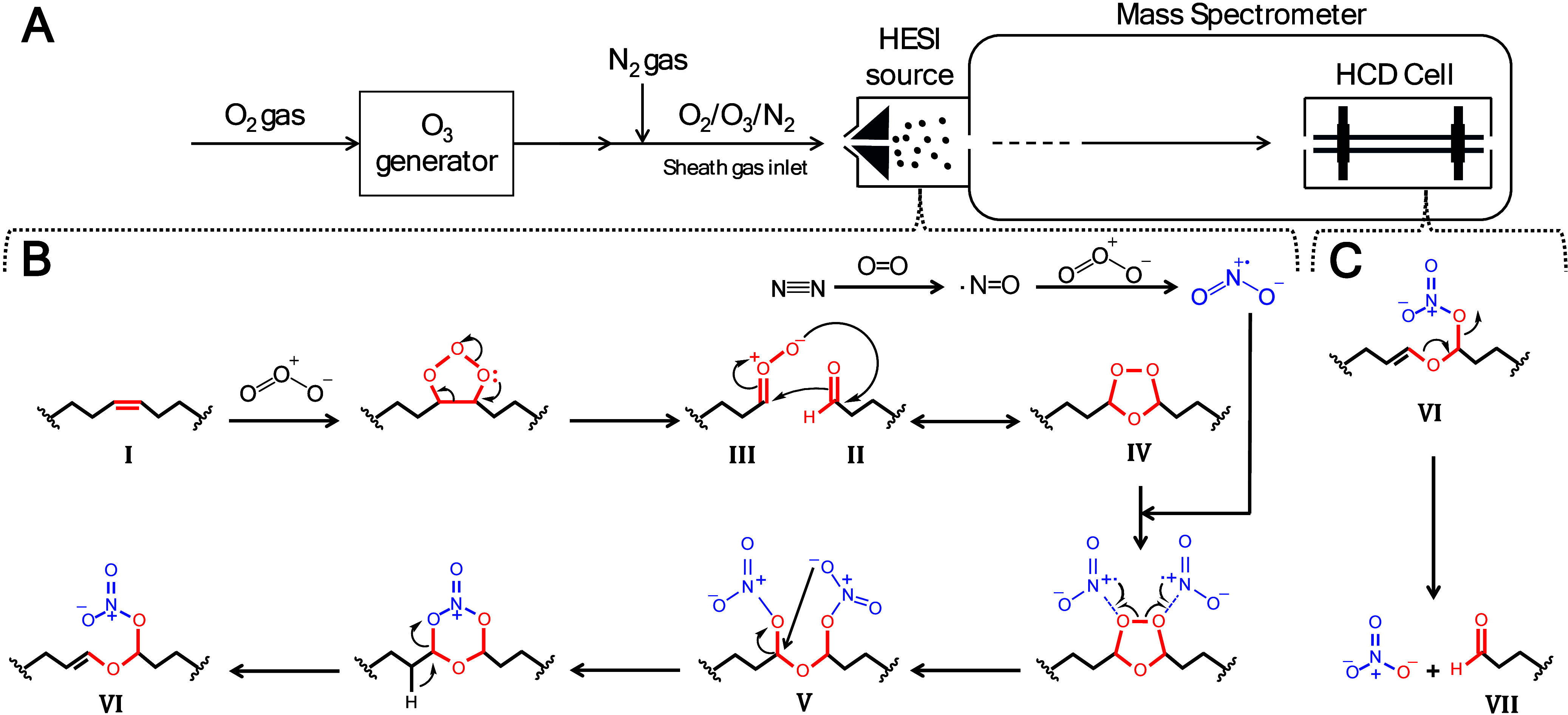
OzNOxESI setup (A), gas-phase reaction mechanism (B), and HCD fragmentation (C). Sheath gas in the MS HESI source was modified to contain oxygen, nitrogen, and ozone. Nitric oxide is generated in situ in a HESI source. Nitrogen dioxide is then generated by the reaction of nitric oxide with ozone. Unsaturated lipids (I) react with ozone to form ozonolysis aldehydes (II) and Criegee intermediates (III). The latter two exist in a brief equilibrium with secondary ozonides (IV). Two NO_2_ molecules attack the secondary ozonide (IV) forming the dominant OzNOx adduct (V). Intramolecular nucleophilic attack leads to the formation of the dominant OzNOx adduct (VI) in positive mode MS. HCD MS^2^ of the second OzNOx adduct (VI) generates product ions (VII) analogous to the ozonolysis aldehydes (II), both of which are diagnostic of the C=C position but here with several distinct advantages by being produced and observed at the MS^2^ level rather than MS^1^.

The OzNOxESI ion chemistry was discovered by noticing the generation of unique [M+NO_4_–H]^+^ and [M+N_2_O_7_]^−^ MS^1^ adducts when unsaturated lipids were exposed to an OzESI setup (Figures 1A and S1), where ozone was mixed with the nitrogen sheath gas in the heated electrospray ionization (HESI) source. These OzNOx species were observed exclusively when the sheath gas contained nitrogen, oxygen, and ozone. Experiments with oxygen and ozone without nitrogen did not produce them, nor did experiments with oxygen and nitrogen without ozone. HCD-MS^2^ spectra of the [M+N_2_O_7_]^−^ ions were dominated by a NO_3_^–^ MS^2^ product ion [*m*/*z* 61.99] (Figure S2). MS^2^ spectra of the [M+NO_4_–H]^+^ ions contain product ions diagnostic of the C=C position, and so this adduct became the focus of the study.

The plausible OzNOxESI reaction mechanism is a system of three gas-phase chemistries (Figure 1B): nitrogen oxides are generated *in situ* in the HESI source; unsaturated lipids react with ozone, producing ozonolysis products; and lipid secondary ozonides react with nitrogen dioxide to produce the novel OzNOx adducts. The *in situ* generation of nitric oxide, assumed to be the rate-limiting step of the methodology, requires not only the mutual presence of both nitrogen and oxygen but also critically the high temperature of the HESI source. Nitric oxide subsequently reacts with ozone to produce nitrogen dioxide. The remaining ozone reacts with unsaturated lipids (I) to produce the classical ozonolysis products: aldehydes (II) and Criegee intermediates (III), which exist in momentary equilibria with secondary ozonides (IV).^24−26^ The secondary ozonide (IV) is then attacked by two nitrogen dioxide molecules producing the [M+N_2_O_7_]^−^ adduct (V), which is the dominant OzNOx adduct observed in negative mode but does not fragment into C=C diagnostic product ions. The [M+N_2_O_7_]^−^ adduct undergoes intramolecular nucleophilic attack to the removal of one NO_3_^–^ moiety, and subsequent hydrogen abstraction yields [M+NO_4_–H]^+^ (VI). This is the dominant OzNOx adduct observed in positive mode and does fragment into C=C diagnostic product ions (VII) (Figure 1C).

The established radical nitrogen oxide chemistry with lipids supports but cannot completely confirm the structure and mechanism of the OzNOx adducts, which due to their novelty must for now remain presumptive.^30^ The final MS^2^ OzNOx product ion (VII) is analogous, sometimes identical, to the ozonolysis aldehyde (II) but has the advantage of being produced at the MS^2^ level in the collision cell rather than in the ionization plume. This difference enables unambiguous assignment of the product ion and the C=C it elucidates to its precursor ion, the lipid that contains the C=C. Figure S3A illustrates a representative negative mode OzNOxESI-MS^1^ spectrum of FA 18:1(*n*-9). It is while studying OzESI of fatty acids that the OzNOxESI chemistry was discovered. We found in our OzESI implementation that the aldehyde intensity (II) was much lower than expected and eventually correlated this to the novel [M+N_2_O_7_–H]^−^ adduct (V). Figure S3B illustrates a representative positive mode OzNOxESI-MS^1^ spectrum of d5-PC 17:0/22:4(*n*-6,9,12,15) with noticeable yield of OzNOx adduct (VI), which is coeluting with the precursor ion at the LC scale (Figure S3C), indicating this reaction is fast enough for coupling with LC. A key difference between OzESI and OzNOxESI is noted. Ozone can attack any of the C=C positions, resulting in four unique ozonolysis aldehydes (II) with four *m*/*z* values in OzESI. The OzNOx adduct (VI), although formed from each of the four C=C, is an ensemble with a singular *m*/*z* value, shared by all four C=C.

### Optimization of the OzNOxESI Workflow

As the OzNOxESI ion chemistry takes place in the HESI ionization plume, source parameters were optimized to maximize the OzNOx ion yield. Methodology and results of stepwise parameter optimization are in Figure S4. Ion transfer tube (capillary) temperature was found to be the most important parameter, with OzNOx ion intensity increasing by an order of magnitude from 200 to 350 °C, supporting the hypothesis that the temperature-dependent *in situ* generation of nitric oxide is the rate-limiting step of OzNOxESI. The optimum auxiliary gas temperature and spray voltage were found to be 350 °C and 3.75 kV, respectively. Through these adjustments, we were able to achieve ∼10% (normalized to the lipid precursor ion) OzNOx adduct ion yield. Optimization of the HCD collision energy was carried out on a class-by-class basis to maximize C=C product ions (Figures S5–13 and Table S1). Optimum collision energy varied greatly by class, such as normalized collision energy (NCE) 10 for fatty acids (Figure S13). Conversely, the MS^2^ spectra of phosphatidylethanolamines (PE) species at low energies (NCE 10–15) mainly showed product ions related to the loss of the headgroup (Figure S6). Higher energies (NCE 20–25) resulted in useful product ions due to further fragmentation at the site of C=C derivatization (Figure S6). Similar trends were observed in phosphatidylcholines (PC), lyso-PE (LPE), phosphatidylglycerols (PG), lyso-PG (LPG), phosphatidylinositols (PI), phosphatidylserines (PS), and phosphatidic acids (PA) (Figures S5, S7–12).

### OzNOxESI Ion Chemistry of Glycerophospholipids (GPLs)

**Figure 2:**
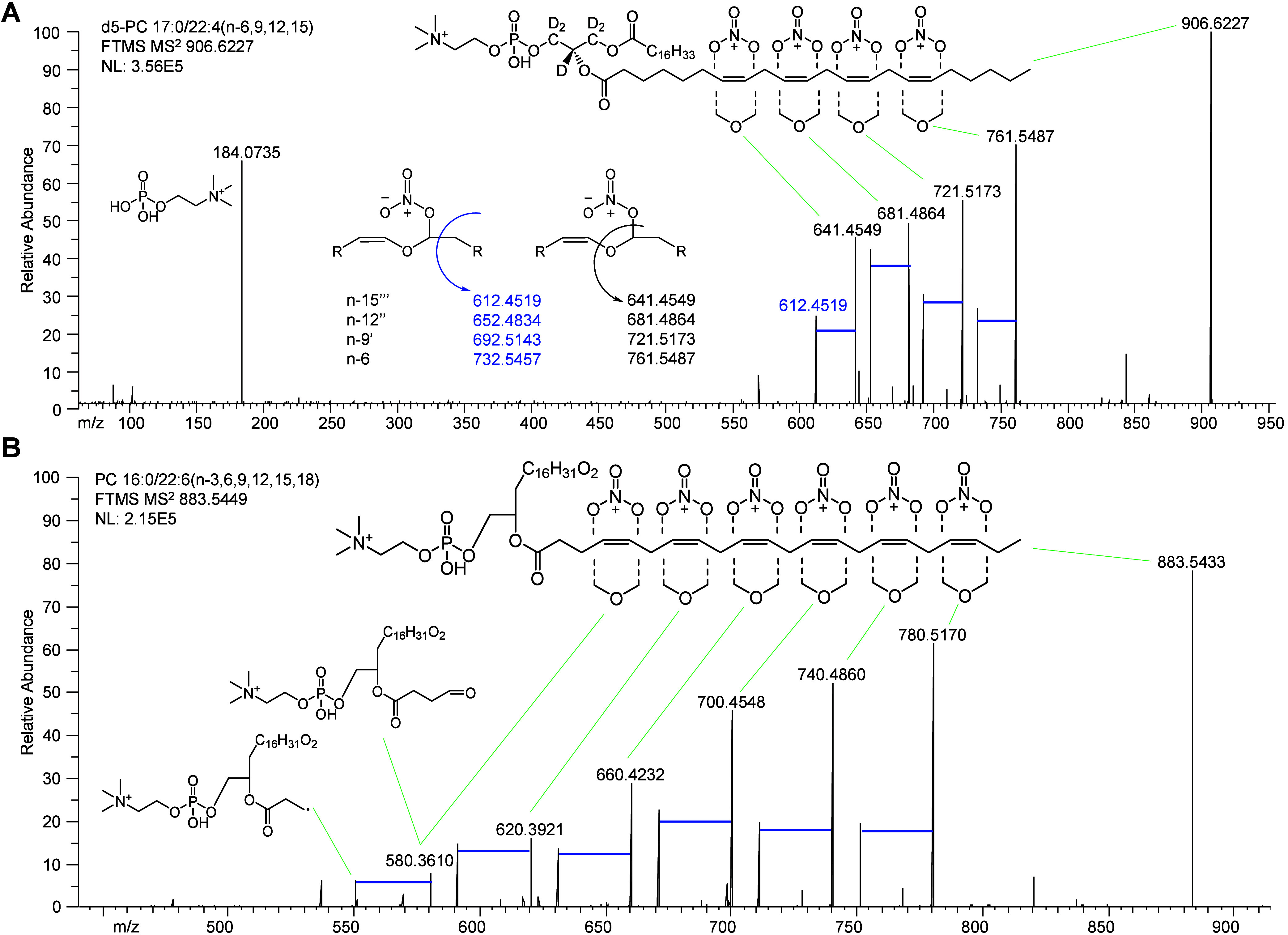
LC-OzNOxESI-MS^2^ spectra of representative PC species. (A) OzNOx HCD-MS^2^ of internal standard d5-PC 17:0/22:4(*n*-6,9,12,15). For each of the 4 C=C, there is a product ion (black) of identical *m*/*z* to the corresponding OzESI-MS^1^ ozonolysis aldehyde and a companion product ion (blue) corresponding to the loss of CHO. (B) OzNOx HCD-MS^2^ spectra of PC 16:0/22:6 from human plasma. Analysis showed this species to be PC 16:0/22:6(*n*-3,6,9,12,15,18). As with the standard, there are a pair of product ions for each C=C in the fatty acyl.

The utility of OzNOxESI for annotating lipid C=C positions was investigated by analyzing ultimateSPLASH standards. We first studied PC lipids, such as *d*_5_-PC 17:0/22:4(*n*-6,9,12,15) (Figure 2A). From the precursor ion ensemble formed from all C=C, a pair of OzNOx-MS^2^ product ions emerges for each C=C: the ozonolysis aldehyde product and a companion product ion (loss of CHO) from a radical fragmentation mechanism. The nature of the radical fragmentation mechanism has several possibilities, one being attack by an oxygen radical and another being charge-remote fragmentation.^13,31,32^ The endogenous PC lipids analyzed using the OzNOxESI workflow behave similarly. For example, LC-OzNOxESI-MS^2^ of PC 16:0/22:6 in human plasma also produced a pair of product ions for each C=C (Figure 2B). In plasma, PC 16:0/22:6(*n*-3,6,9,12,15,18) was found to be the predominant C=C regioisomer. It is worth mentioning that the signal intensity of its OzNOx-MS^2^ product ions decreases as the C=C location moves further up the fatty acyl chain (Figure 2). This pattern was far less pronounced in an equal molar mixture of monounsaturated PC 18:1/18:1, where the diagnostic ion intensity ratio of the n-7, n-9, n-10 and n-12 fatty acyls is 1.0/0.86/0.80/0.72 (Figures S14), and not observed across all polyunsaturated lipids belonging to other classes (e.g., PE) where the product ions were often of approximately equal signal intensities. This might be due to the different collision energies utilized for different lipid classes or the steric hindrance exerted by certain head groups. As for lysoPC (LPC), LPC 16:1 in human plasma was found to be a mixture of several C=C regioisomers (Figure S15). The difference in intensity between MS^2^ product ions enables the relative quantification of these isomers. This relative quantification can be translated into concentrations by absolute quantification of the lipid LC-MS feature and partitioning the absolute concentration across regioisomers according to their relative product ion abundances.

**Figure 3:**
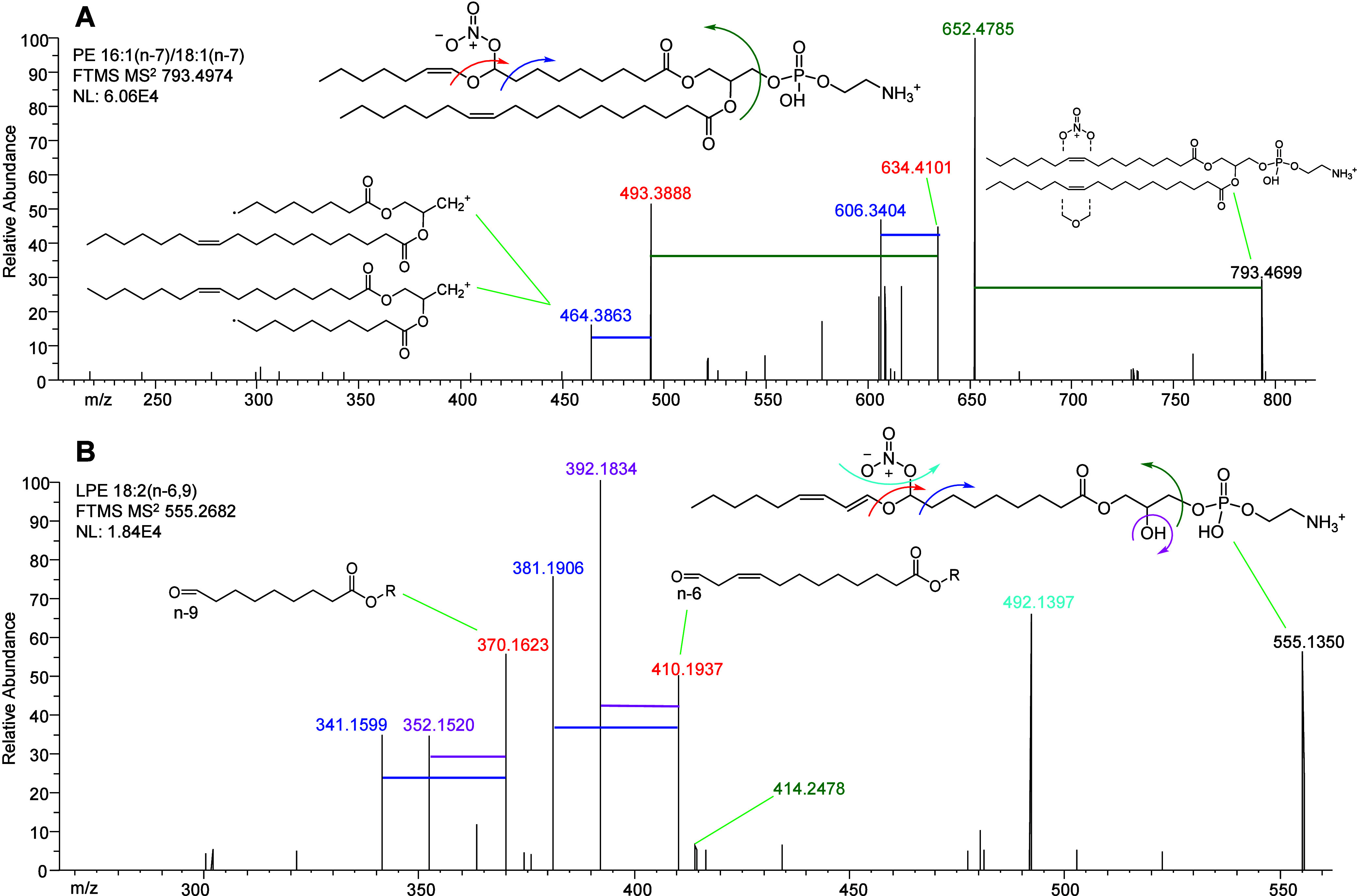
LC-OzNOxESI-MS^2^ spectra of representative (L)PE species. (A) OzNOx HCD-MS^2^ spectra of PE 16:1_18:1 from human plasma. Analysis revealed this species to be predominately PE 16:1(*n*-7)_18:1(*n*-7). While this lipid has two C=C, their products have the same *m*/*z*. There are paired *m*/*z* for the C=C positions, detectable with and without the headgroup. (B) OzNOx HCD-MS^2^ of LPE 18:2 from human plasma. Analysis revealed this species to be predominately LPE 18:2(*n*-6,9). The C=C product ions are triplets with a loss of H_2_O (purple).

OzNOxESI was similarly tested with PE standards, such as *d*_5_-PE 17:0/22:4(*n*-6,9,12,15) (Figure S16). While PE undergoes the same OzNOx derivatization as PC, LC-OzNOxESI-MS^2^ spectra for PE illustrate a different fragmentation scheme involving the loss of the headgroup (Figure 3). This difference between PE and PC fragmentation is in part due to the use of different collision energies (NCE 25 and NCE 15, respectively). The loss of the headgroup is followed by fragmentation at the site of C=C derivatization. Because of the headgroup loss, the PE product ions are analogous but not identical to OzESI ozonolysis aldehydes. Endogenous PE 16:1_18:1 (Figure 3A) in human plasma followed the similar MS^2^ fragmentation behavior as the PE standard. LC-OzNOxESI-MS^2^ showed that this species is predominately PE 16:1(*n*-7)_18:1(*n*-7) in human plasma. As these two C=C have the same *n*-# position in their fatty acyl chains, their product ions are of identical mass, and so only one set of *m*/*z* values is observed. The PC product ion pairing is repeated in PE but duplicated with and without the loss of the headgroup (Figure 3A). As for lysoPE (LPE) lipids, OzNOx-MS^2^ has a fragmentation pattern different from two-tailed PE as the loss of the headgroup is much reduced (Figure 3B). This is likely due to the difference in collision energies, NCE 15 for LPE and 25 for PE. Lyso species generally have two fragmentation events not broadly seen in two-tailed GPLs: the loss of the NO_3_^–^ moiety and the loss of H_2_O. LC-OzNOxESI-MS^2^ of endogenous LPE 18:2 revealed a product ion trio for each C=C: one identical to the OzESI ozonolysis aldehyde, one with the loss of water, and one following the radical fragmentation mechanism (Figure 3B). This plasma lipid was predominantly the *n*-6,9 isomer (Figure 3B and Table S2).

LC-OzNOxESI-MS^2^ of other GPL classes (i.e., PG, PI, and PS) is depicted in Figure S17. At their optimized collision energies—25, 23, and 23, respectively—these classes follow the same fragmentation pattern as PE with the loss of the headgroup. Because of this, PE, PG, PI, and PS species that have the same fatty acyl composition yield the same OzNOx-MS^2^ product ions. This may be useful when normalizing glycerophospholipid C=C characteristic product ions to shared *m*/*z* values. Also noteworthy is that this unity is achieved regardless of the original adduct type; [M + H]^+^, in the case of PE and PS, or [M+NH_4_]^+^, for PG and PI species. Regardless of lipid adduct, they arrive at the same product ions specific to their C=C positions in *m*/*z* independent of the headgroup. OzNOx-MS^2^ spectra of two additional GPL classes, LPG and PA, can be seen in Figure S9 and Figure S12, respectively. These classes follow the same pattern at their collision energies (NCE = 23 and 29, respectively).

### OzNOxESI Ion Chemistry of Fatty Acids (FA)

**Figure 4:**
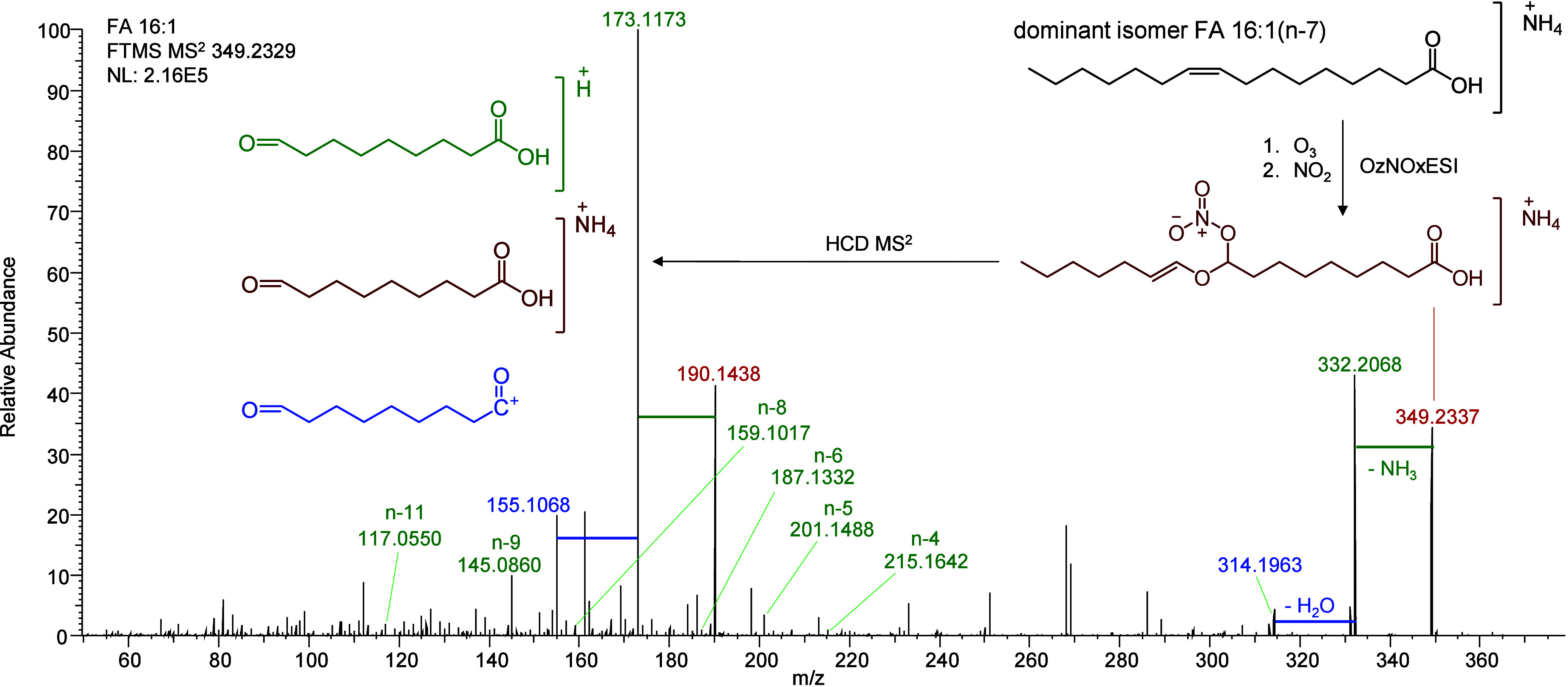
OzNOx HCD-MS^2^ of FA 16:1 from human plasma. A trio of product ions are observed per C=C regioisomer with successive loss of the ammonium adduct and then H2O. FA 16:1 was found here to primarily be FA 16:1(*n*-7), but a variety of lesser regioisomers were also detected. While the proton adduct (green) is the most intense here, the ammonium adduct (red) was the species used in data processing, as it was more consistently observed over a variety of fatty acid chain lengths and degrees of unsaturation.

While fatty acids are typically analyzed in negative mode, negative mode HCD fragmentation of the OzNOx adduct has not shown product ions diagnostic of the C=C position. Fortunately, positive mode OzNOx-MS^2^ yields C=C specific product ions for FA. For example, when analyzing standard FA 18:1(*n*-9), three ozonolysis-related product ions were generated as [M+NH_4_]^+^, [M+H]^+^, and [M+H–H_2_O]^+^. The highest intensity of these three was observed at NCE 10, the minimum collision energy on the Q Exactive HF (Figure S13). OzNOx-MS^2^ of oleic acid (FA 16:1) in human plasma showed 7 C=C regioisomers. The predominant member was the *n*-7 isomer (92.1%), followed by *n*-9 (6.9%). *n*-5, *n*-6, *n*-10, *n*-11, and *n*-12 were also identified but at low abundances (combined ∼1%) (Figure 4 and Table S3).

### Comparing the Classical OzESI and Novel OzNOxESI Workflows

**Figure 5:**
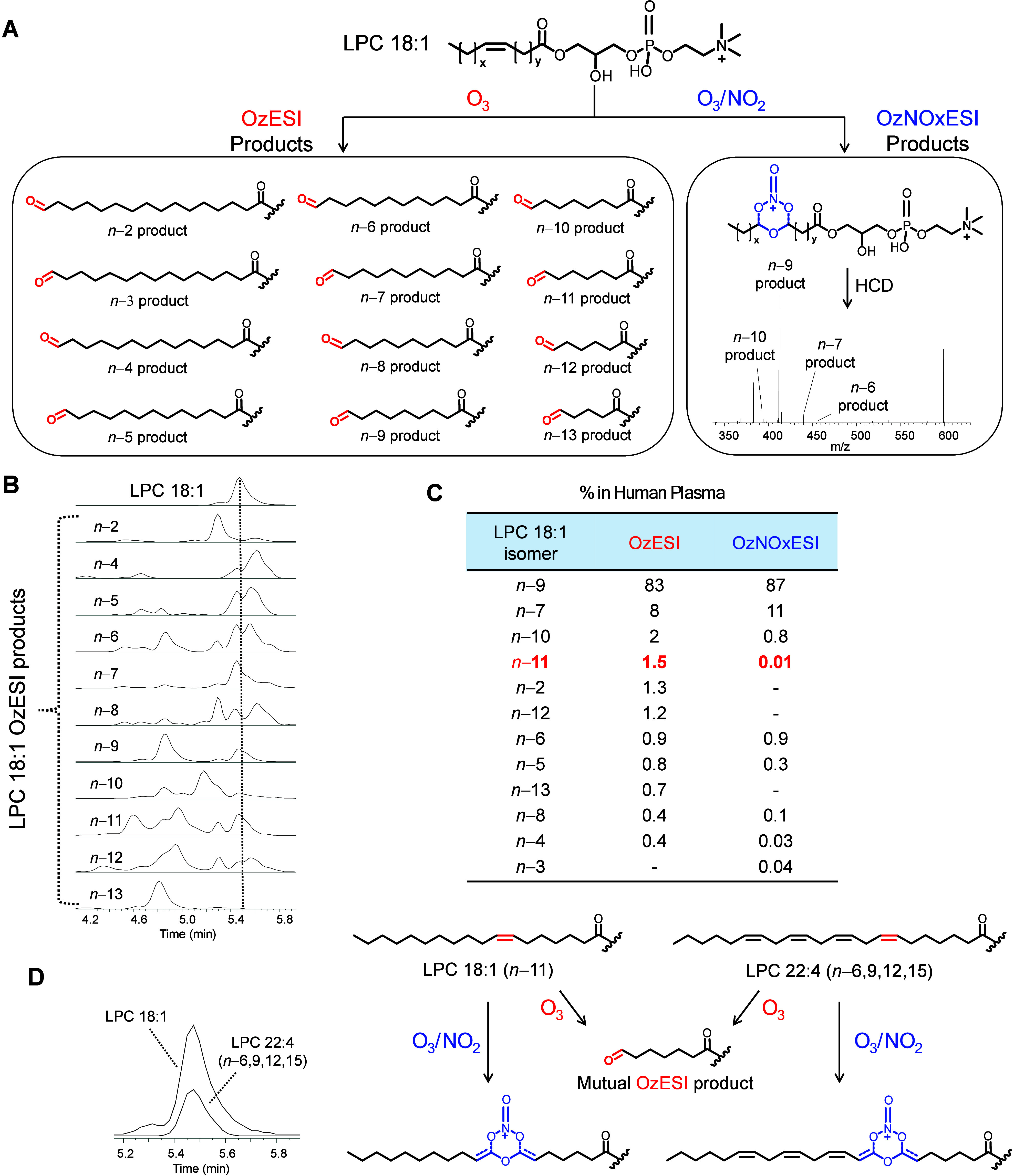
Comparing LC-OzESI-MS^1^ and LC-OzNOxESI-MS^2^ workflows using LPC 18:1 in human plasma. (A) Ozonolysis products detected by OzESI or OzNOxESI in the same run. The major OzESI-MS^1^ and OzNOxESI-MS^2^ product ions are identical for LPC, differing only in the places they are observed: OzESI products as LC-MS features and OzNOxESI product ions at the MS^2^ level after mass selection of the OzNOx adduct. (B) OzESI EIC of LPC 18:1 and OzESI products detected in the same scan as the maximum LPC 18:1 intensity (dotted line). Poor alignment of OzESI products and multiple peaks is due to other LPC lipids with adjacent or overlapping RT. (C) Comparing LPC 18:1 C=C regioisomer quantification by OzESI and OzNOxESI. Highlighted is LPC 18:1(n–11), inflated ∼150x in OzESI vs OzNOxESI. (D) Illustrative example of the OzESI coelution challenge and explanation of OzESI’s inflated quantification of LPC 18:1(n–11) in this data set.

The precursor lipid coelution issue of OzESI and how OzNOxESI overcomes this challenge can be illustrated using endogenous LPC 18:1 in human plasma (Figure 5). In OzNOxESI, both classical OzESI and novel OzNOxESI product ions (Figure 5A) can be observed in a single LC-MS/MS analysis. At the MS^1^ level, LPC 18:1 had a well-defined peak (Figure 5B). However, the peaks of its theoretical OzESI ozonolysis aldehydes were, in many cases, not well-aligned with the precursor peak (Figure 5B). This is because the theoretical ozonolysis aldehydes are shared by other LPC species with overlapping or neighboring LC-MS peaks. The coelution of other LPC lipids creates ambiguity in assigning MS^1^ OzESI aldehydes to precursor lipids. In OzNOxESI, the ambiguity is eliminated by mass selection prior to dissociation into product ions. With mass selection, the OzNOx-MS^2^ spectrum of LPC 18:1 contains only C=C product ions from LPC 18:1 (Figure 5A, right). Comparing results from the same LC-MS injection, the OzESI and OzNOxESI approaches agreed on the top two isomers (*n*-9 and *n*-7) and had similar quantification of them (Figure 5C). However, the OzESI data suggested the existence of a greater number of regioisomers with minor abundances, which is presumably due to LPC coelution. Most dramatically, the OzESI data suggested an *n*-11 isomer at 1.5% abundance, which is 150 times higher than the OzNOxESI abundance of the same isomer (Figure 5C). The source of *n*-11 inflation is the coelution of LPC 22:4 and LPC 18:1 in this data set (Figure 5D). The OzNOx-MS^2^ spectrum of LPC 22:4 showed that the predominant isomer of this species was *n*-6,9,12,15. The *n*-15 C=C of LPC 22:4(*n*-6,9,12,15) and the C=C of LPC 18:1(*n*-11) yield identical OzESI ozonolysis aldehydes, leading to ambiguity in regioisomer assignment and inflating perceived LPC 18:1(*n*-11) abundance in OzESI (Figure 5D). While this example illustrates the superiority of OzNOxESI to classical OzESI, the fact that both sets of product ions can be observed and analyzed in the same LC-MS run is of great interest in untangling the complexity of coelution and isomerism in LC-MS based lipid analysis.

### OzNOx Companion for Semiautomated Analysis of OzNOxESI Data

Software solutions^3,33^ exist for processing LC-MS lipid ozonolysis data, but they are currently incompatible with OzNOxESI due to its novel chemistry. Therefore, we developed a new data processing solution: OzNOx Companion (Figure S18). Briefly, OzNOx Companion generates exhaustive lists of theoretical OzESI and OzNOxESI ozonolysis product *m*/*z* values using an input list of lipid annotations and searches LC-MS data for OzESI-MS^1^ and OzNOx-MS^2^ product ions. OzNOx Companion lists the possible regioisomers of each lipid species through exhaustive combinatorial pairing of OzNOx-MS^2^ product ions. For monounsaturated lipids, the number of regioisomers is equal to the number of detected OzNOx-MS^2^ product ions. For polyunsaturated lipids, the number of theoretical regioisomers can significantly surpass the number of OzNOx-MS^2^ product ions due to combinatorial expansion with more C=C per molecule. Fortunately, many combinations can be eliminated based on incompatibility. For example, FA 22:4(*n*-6,9,12,15) produces four unique product ions, each indicating the position of one C=C. Each product ion also indicates the number of up- and/or downstream C=C: the *n*-9 *m*/*z* suggests that there is one C=C at lower *n*-# and two C=C at higher *n*-#. Furthermore, each successive C=C must be at a higher *n*-# than that before it. Such considerations provide logic for chaining C=C and invalidating many combinations. OzNOx Companion carries out probability scoring of logical theoretical regioisomers on a scan-by-scan basis, utilizing aligned spectra when there is replicate sampling. OzNOx Companion uses the probability scores and several mathematical assumptions to divide the total signal intensity of a lipid among its plausible regioisomers. These mathematical assumptions include neglecting species projected to be below 1% relative abundance where necessary and that C=C diagnostic ion intensity is independent of C=C position on the fatty acyl chain. For coeluting polyunsaturated lipid isomers, the C=C diagnostic ion unique to each isomer is the basis for quantification, which is typically the highest abundance ion for that isomer as it is often close to the methyl end. OzNOx Companion reports the OzESI-MS^1^ products, OzNOxESI-MS^2^ products, theoretical regioisomers, and relative quantification of confirmed regioisomers. For more information on the OzNOx Companion and example files, see the Data and Code Availability.

### OzNOxESI-Based LC-MS Analysis of Human Plasma

To further test OzNOxESI, the workflow was applied to lipid extracts from human plasma, focusing on unsaturated GPLs. First, pooled plasma samples were analyzed in positive and negative modes without exposure to ozone and oxygen. The collected spectra were analyzed by using LipidSearch and MS-DIAL to annotate lipids. Next, LC-OzNOxESI-MS^2^ data were collected in PRM mode using target lists generated by OzNOx Companion. The PRM spectra were then analyzed using OzNOx Companion to detect OzNOx-MS^2^ product ions and annotate lipid C=C regioisomers. Results include 270 endogenous unsaturated GPL regioisomers identified and quantified in human plasma, including 193 PC, 5 PE, 8 PI, 53 LPC, and 11 LPE species (Table S2). All GPLs were quantified against ultimateSPLASH deuterated standards spiked before extraction. Analysis of the LC-OzNOxESI-MS^2^ data resulted in correctly annotating the spiked ultimateSPLASH standards of PC, PE, PG, PI, and PS classes (Tables S4–5), demonstrating the ability of the OzNOxESI workflow to accurately annotate lipid C=C in the presence of a complex biological matrix, such as plasma. For GPLs carrying monounsaturated fatty acyls, in most cases, *n*-7 (for C16:1), *n*-8 (for C17:1), and *n*-9 (for C18:1) were the most abundant (Figure S19). For diunsaturated GPLs, *n*-6,9 was the predominant isomer (Figure S19). GPLs having triunsaturated fatty acyl chains mainly contained C18:3 or C20:3 fatty acyls. For the C18:3 fatty acyls, *n*-3,6,9 was the predominant isomer (Figure S19). However, *n*-6,9,12 was the predominant isomer for GPLs with C20:3 fatty acyl chains. In the case of PC(18:0_20:3), the major isomer was identified at RT 15.0 min, which mainly contained the *n*-6,9,12 regioisomer (99.5%). However, a minor isomer peak was detected at RT 15.6 min, a *n*-9,12,15 regioisomer (97%). In GPLs with fatty acyls having 4 C=C, species with C20:4 arachidonic acid (*n*-6,9,12,15) or C22:4 adrenic acid (*n*-6,9,12,15) were the most abundant. Furthermore, GPLs bearing polyunsaturated fatty acids (PUFAs) with 6 C=C predominantly included C22:6 docosahexaenoic acid (*n*-3,6,9,12,15,18). Some minor species (e.g., PC 37:3 at RT 13.9 min) lacked sufficient MS^2^ data in negative mode to annotate the molecular species composition and were reported with summed acyl composition. However, OzNOxESI yielded reliable product ions, enabling annotation of the C=C positions to be predominantly *n*-6,9,12.

The plasma total fatty acid content was also analyzed (Table S3). Hydrolyzed lipid extract from human plasma was subjected to LC-MS analysis in negative mode. Eighteen fatty acids were detected based on accurate mass and RT alignment with known standards (Table S6). The absolute concentrations of the identified fatty acids are comparable to those reported in the literature.^34^ OzNOxESI enabled the identification and quantification of 36 fatty acid C=C isomers. Among them, *n*-7 (for FA 16:1), *n*-8 (for FA 17:1), and *n*-9 (for FA 18:1) were the most abundant. In the case of PUFAs, isomers with methylene-interrupted C=C were the most abundant (e.g., FA 20:3 *n*-6,9,12, FA 20:5 *n*-3,6,9,12,15 and FA 22:6 *n*-3,6,9,12,15,18). The distribution of fatty acid C=C regioisomers in total fatty acids largely agrees with the results of GPL analysis (Figure S19) and with the literature,^3,33^ confirming the applicability of OzNOxESI to elucidate C=C regioisomerism in lipids even in complex biological matrices with simplicity of execution and confident annotation.

## Discussion

Tracking shifting lipid C=C isomerism in lipids not only sheds light on the complexity of the biological lipidome but also enables the detection of minor yet significant perturbations in lipid isomer ratios caused by certain disease conditions. Despite the significant progress made to enable resolution of lipid C=C positions, the existing technologies have their limitations, often including mass spectrometers with unique fragmentation capabilities, in solution sample preparation before analysis, or specialized hardware, such as ion mobility equipped instruments. As one of the promising methods for C=C annotation, online ozonolysis, when connected to the front of mass spectrometers, can provide highly efficient ozonolysis for effective coupling with LC separation but suffers in its ability in unambiguous assignment of C=C to its original lipid precursor when multiple lipids are co-ionized in the ionization plume.

The current study aimed to provide a more accessible solution to the practical obstacles of C=C ozonolysis. We discovered and rigorously tested a novel ozonolysis pathway capable of unambiguously annotating lipid C=C positions. The newly discovered ozonolysis products were only formed upon exposure of lipid molecules to a mixture of nitrogen, oxygen, and ozone inside the ion source region of a mass spectrometer. Our previous OzESI experiments using similar settings did not yield the new OzNOx adducts without nitrogen. That suggested the involvement of nitrogen in generating these adducts whose exact masses and MS^2^ spectra implied the presence of nitrogen oxides. Atmospheric nitrogen oxides mainly arise as the product of terrestrial pollution but nitric oxide can also be created from nitrogen and oxygen at high temperature by jet engines,^35^ which is why heating in the ionization source is needed. The nitric oxide subsequently reacts with ozone to produce nitrogen dioxide. The latter reaction is widely used in ozone-based chemiluminescence detection of nitrogen oxides in different fields such as environmental analysis.^36^ In the present study, it is proposed that *in situ* generated nitrogen dioxide reacts with conventional secondary ozonides, yielding the new OzNOx adduct. This is supported by previous studies reporting the reaction of unsaturated fatty acids with nitrogen dioxide yielding allylic nitro and nitrite derivatives of linoleate.^30^ The MS^2^ spectra of the OzNOx adducts revealed fragment ions that are informative of C=C position patterns and the presence of unique patterns that are lipid-class specific and, in some classes, collision-energy selective. A semiautomated workflow was developed alongside OzNOx Companion for the stepwise analysis of conventional untargeted LC-MS/MS lipidomics data, the generation of target list for performing OzNOx-MS^2^, and finally description of lipid C=C regioisomerism with high sensitivity and specificity, demonstrated by the correct annotation of deuterated GPLs spiked into plasma matrix and further demonstrated in the resolution of 270 GPL and 36 fatty acid C=C isomers when applying the OzNOx workflow for the analysis of human plasma.

We anticipate that this novel workflow can be readily applied to deciphering lipidome complexity due to C=C regioisomerism in other matrixes such as tissues and cells. While this study focused on GPLs, further development is needed to elucidate C=C in sphingolipids and glycerolipids, where stereo hindered C=C and more complex structural isomerism are dominant. Another limitation of this study is its relative quantification of coeluting lipid C=C regioisomers. Currently, it is based on the assumption that the C=C position does not affect the respective position-diagnostic ions. While this seems true for the PE, PG, PI and PS lipids, and close to true for monounsaturated PCs (Figure S14), a clear decline in intensity is observed when C=C is moving away from the methyl end on fatty acyls in polyunsaturated PCs (Figure 2). Future work is needed to derive a reliable distribution model in quantification of coeluting unsaturated PC isomers when more C=C position specific PC standards become commercially available.

## Experimental Section

### Materials

Lipid standards were acquired from Avanti Polar Lipids (Alabaster, AL). Fatty acid standards were acquired from Avanti Polar Lipids (Alabaster, AL) and Cayman Chemical (Ann Arbor, Michigan). Solvents and reagents were acquired from ThermoFisher Scientific (Atlanta, GA).

### OzNOxESI Instrumentation

All MS experiments were performed on a Q Exactive HF mass spectrometer (ThermoFisher Scientific). The minimal MS ion source modification necessary for OzNOxESI is shown in Figure S1A. Titan-30 ozone generator (Absolute Ozone, Edmonton, Canada) was operated at an oxygen gas flow of 0.25 mL/min and ∼7% ozone output as measured by an in-line 106-H ozone monitor (2B Technologies, CO). Ozone was introduced to the nitrogen sheath gas line by the T-junction. The gas mixture that reached the HESI probe was approximately 53% nitrogen, 44% oxygen, and 3% ozone. The ion source was vented through a canister containing an ozone destruction catalyst. The entire setup was monitored for ozone leak with a 106-L ozone monitor (2B Technologies) with ambient ozone concentration below 0.1 ppm throughout the analyses.

### Lipid Extraction

Lipids from pooled human plasma were extracted using a modified MMC method with ultimateSPLASH lipid standards spiked in as previously reported.^37,38^ Extracted lipids were resuspended in 60 μL of ACN/IPA/H_2_O (65:30:5, *v*/*v*/*v*) and then subjected to LC-MS analysis.

### Total Fatty Acid Preparation

Lipids were extracted from human plasma (10 μL) by protein precipitation using isopropyl alcohol and hydrolyzed by 0.3 M KOH in MeOH:H_2_O (80:20) to obtain total fatty acids via a previously published method.^39^ The total fatty acids were resuspended in 100 μL of IPA/EtOH/ACN (1:1:1, *v*/*v*/*v*) and subjected to LC-MS analysis.

### LC-MS Conditions

All LC-MS analyses were carried out using a Vanquish Horizon UHPLC instrument (ThermoFisher Scientific) coupled to the aforementioned mass spectrometer. Separation of lipids was performed on an ACQUITY Premier CSH C18 column (100 mm × 2.1 mm, 1.7 μm, Waters). A mobile phase composition and gradient system was reported previously.^37^ Data for untargeted lipidomics were collected using Full-MS followed by data-dependent HCD-MS/MS in positive and negative modes. The spectral acquisition for total fatty acid extracts utilized Full-MS in the negative mode. OzNOxESI data were collected under positive mode parallel reaction monitoring (PRM). The MS parameters for untargeted lipidomics were reported elsewhere.^37^ The MS acquisition parameters for OzNOxESI were similar to that of untargeted lipidomics except for sheath gas flow rate of 10 Arb to prevent backflow of nitrogen to the ozone generator and HESI parameters discussed previously. Mass resolution was 30k (at 200 *m*/*z*). The AGC target was set as 1e^5^ with a maximum injection time of 75 ms. HCD collision energies are given in Table S1.

### Untargeted Lipidomics Data Analysis

Lipidomics data was processed using LipidSearch (version 5.1.8, Thermo Scientific) as described previously.^37^ Only lipid molecular species with CV < 25% in QC samples were kept and the identified lipids information (molecular formula, RT, and ion adduct type) was converted to a precursor library for MS-DIAL^40^ (version 4.9.221218) for accurate quantification of area under the curve (AUC) of each lipid hit. For quantification, AUC values were first corrected for ^13^C abundance (Type I correction) following Lipidomics Standards Initiative^41^ guideline and the practice of others.^42,43^ The absolute concentrations of identified lipids were calculated using ionization response factors derived from spiked deuterated standards (Table S5).

### Total Fatty Acids Data Analysis

Total fatty acids data were processed using Skyline^44^ (version 24.1). The fatty acid peaks were autointegrated by searching RAW files against a pseudotransition list containing an exhaustive list of fatty acids with accurate mass and retention time determined either by using fatty acid standards or by a RT prediction model. Fatty acid concentrations were determined from calibration curves of each analyte, which were constructed by normalizing to the selected deuterated internal standards (Table S7) followed by linear regression of external standards (Table S6) with 1/*x* weighting.

### Direct Infusion for OzNOxESI Optimization

Direct infusion by a Hamilton (Reno, NV) syringe pump was conducted for the HESI source parameter and HCD collision energy optimization while imitating the lipidomics methodology. Standards were diluted in 30% mobile phase A and 70% mobile phase B for infusion, where A and B are the mobile phase system of the lipidomics method. The syringe was connected to a PEEK T-junction intercepting LC eluate (also 30% A, 70% B). The combined flow rate of LC and syringe was the same as the lipidomics method, 230 μL/min. Further details of the direct infusion experiments and optimized parameters are in Figures S4–13.

### OzNOxESI Data Analysis

OzNOx Companion was used to validate the RT of annotated lipid species, generate PRM target lists, detect and quantity OzNOx-MS^2^ product ions, and perform the bulk of C=C regioisomer quantification. In some cases, due to the complexity of the detected product ion list and resulting theoretical regioisomers, OzNOx Companion could not assign regioisomer percentages and instead provided the list of theoretical regioisomers and their probability scores. In these cases, manual curation was conducted using the reported OzNOx-MS^2^ product ions and raw data inspection.

## Data Availability

The data that supports the findings of this study are publicly available on MassIVE database under accession code MSV000096085. OzNOx Companion was coded in Python with the copy, datatime, math, matplotlib, numpy, os, pandas, and re packages. The source code, ReadMe, and example files are available on Github (https://github.com/QibinZhangLab/OzNOx-Companion).

## Supporting Information Available

Instrument modification; OzNOx negative-mode MS^2^ spectrum; LC-OzNOxESI positive- and negative-mode MS^1^ spectra; design and results of direct infusion experiments; additional representative LC-OzNOxESI-MS^2^ spectra; OzNOx Companion workflow; lipid-bound acyl vs total fatty acids C=C regioisomerism (PDF)
HCD collision energy and mass loss by class; OzNOx-based annotation of standards; plasma lipids identified; plasma fatty acids identified; lipid internal standards; fatty acid internal standards; fatty acid external regression standards (XLSX)
