# Supplementary material for: OzNOxESI: A Novel Mass Spectrometry Ion Chemistry for Elucidating Lipid Double-Bond Regioisomerism in Complex Mixtures
Source: Anal Chem. 2025 Jan 16;97(3):1879–88. doi: 10.1021/acs.analchem.4c05940 (PMC11780577; doi:10.1021/acs.analchem.4c05940)
Supplement: Supplementary file 1 — ac4c05940_si_001.pdf [file ac4c05940_si_001.pdf]

Supporting Information

OzNOxESI: A novel mass spectrometry ion chemistry for elucidating lipid double-bond regioisomerism in complex mixtures

Ryan A. Smith[1,‡], Ashraf M. Omar[1,‡], Fayaj A. Mulani[1], Qibin Zhang[1,2]*

[1]Center for Translational Biomedical Research, University of North Carolina at Greensboro, North Carolina Research Campus, Kannapolis, NC 28081, USA

[2]Department of Chemistry & Biochemistry, University of North Carolina at Greensboro, Greensboro, NC 27402, USA

[‡]These authors contributed equally

*email: q_zhang2@uncg.edu

Table of Contents:

- **Figure S1:** Addition of oxygen and ozone to nitrogen sheath gas by T-junction
- **Figure S2:** Example negative ion mode OzNOxESI HCD-MS^2 spectrum
- **Figure S3:** Representative LC-OzNOxESI-MS spectra of unsaturated lipids
- **Figure S4:** Stepwise optimization of HESI-II OzNOxESI by eluate infusion of PC standard
- **Figure S5:** HCD optimization for LC-(+)-OzNOxESI-MS^2 analysis of PC lipids
- **Figure S6:** HCD optimization for LC-(+)-OzNOxESI-MS^2 analysis of PE lipids
- **Figure S7:** HCD optimization for LC-(+)-OzNOxESI-MS^2 analysis of LPE lipids
- **Figure S8:** HCD optimization for LC-(+)-OzNOxESI-MS^2 analysis of PG lipids
- **Figure S9:** HCD optimization for LC-(+)-OzNOxESI-MS^2 analysis of LPG lipids
- **Figure S10:** HCD optimization for LC-(+)-OzNOxESI-MS^2 analysis of PI lipids
- **Figure S11:** HCD optimization for LC-(+)-OzNOxESI-MS^2 analysis of PS lipids
- **Figure S12:** HCD optimization for LC-(+)-OzNOxESI-MS^2 analysis of PA lipids
- **Figure S13:** HCD optimization for LC-(+)-OzNOxESI-MS^2 analysis of fatty acids
- **Figure S14:** OzNOxESI-MS2 of equimolar mixture of PC 18:1/18:1 regioisomers
- **Figure S15:** LC-OzNOxESI-MS^2 of LPC 16:1 from human plasma
- **Figure S16:** LC-OzNOxESI-MS^2 of internal standard d_5-PE 17:0/22:4(n-6,9,12,15)
- **Figure S17:** LC-OzNOxESI-MS^2 acquisition of representative PG, PI, and PS species
- **Figure S18:** LC-OzNOxESI-MS/MS workflow and functionality of OzNOx Companion
- **Figure S19:** Distribution of C=C regioisomers in representative fatty acids in human plasma

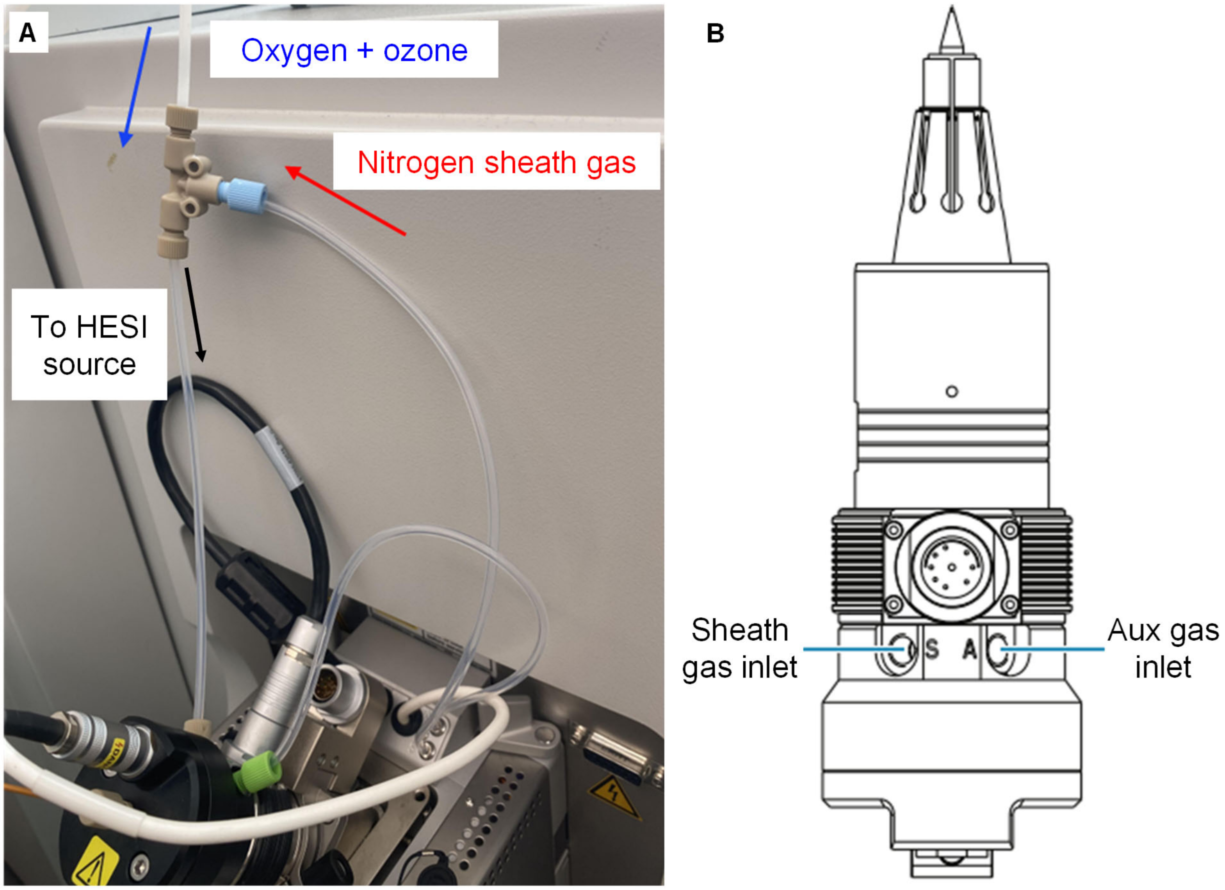

Figure S1. Addition of oxygen and ozone to nitrogen sheath gas by T-junction. (A) Photograph of Thermo HESI-II source docked with Thermo Q Exactive HF mass spectrometer. Marked by the red arrow, the nitrogen sheath gas line normally connects directly from the mass spectrometer to the HESI-II source, now connects to a PEEK T-junction, where the nitrogen gas mixes with oxygen and ozone gases before going to the sheath gas inlet. The oxygen and ozone line connects to an Absolute Ozone ozone generator (not pictured), which is fed by a mixture of 95% oxygen balanced by 5% nitrogen. The ozone generator produces a mixture of ~ 7% ozone in oxygen. After the T-junction, the final gas mixture that reaches the HESI-II source is approximately 53% nitrogen, 44% oxygen, and 3% ozone. (B) Depiction of the HESI-II source provided by Thermo Fisher Scientific in top-down orientation.

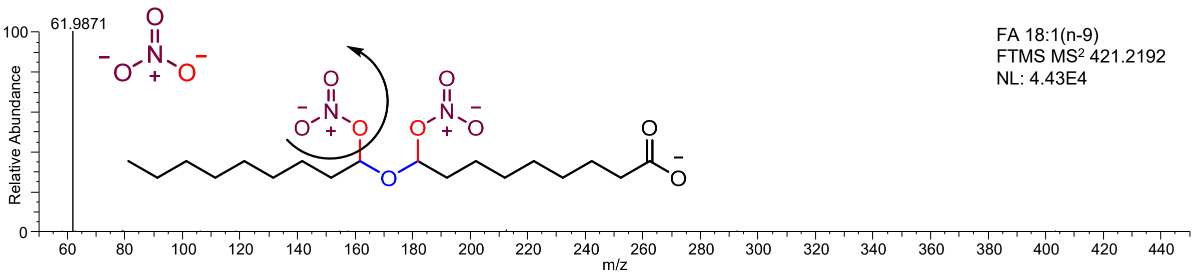

Figure S2. Example negative ion mode OzNOxESI HCD-MS² spectrum in LC-(-)-OzNOxESI-MS² acquisition of FA 18:1(*n*–9). Depicted is the major OzNOxESI adduct found in negative mode for unsaturated lipids surveyed in this work, the C=C replaced by N_2O_7, and its presumptive structure. The major HCD product ion observed is that of the liberated NO_3^- moiety, which may have future application for detection of unsaturated lipids in data-dependent methods but does not indicate C=C positions.

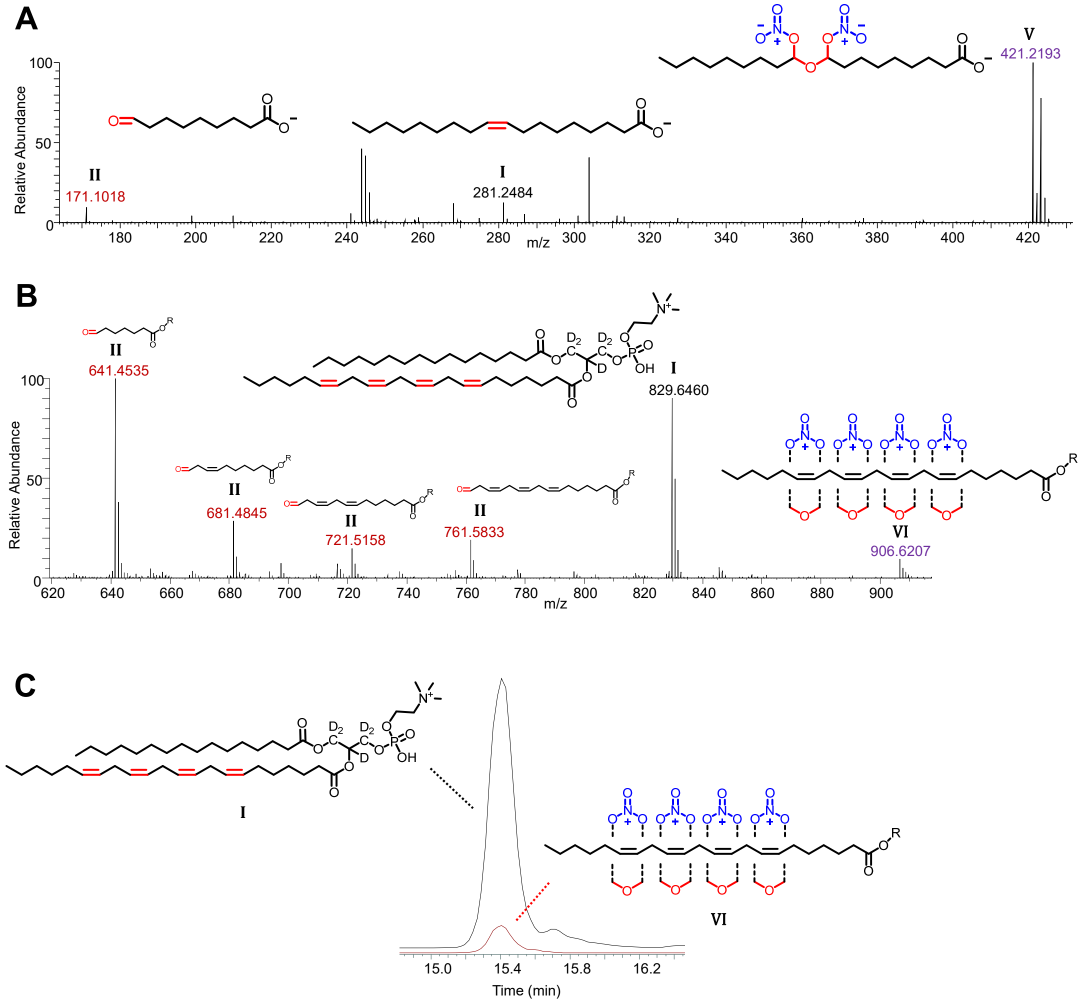

Figure S3. Representative LC-OzNOxESI-MS spectra of unsaturated lipids. (A) Represenatitive LC-(-)-OzNOxESI-MS¹ spectrum with FA 18:1(*n*–9) from fatty acid standard mixture in neat solution. (B) Representative LC-(+)-OzNOxESI-MS¹ spectrum with d5-PC 17:0/22:4(*n*–6,9,12,15) from lipidomics standard mixture in neat solution. (C) Overlayed XICs of $[M + H]^+$ and OzNOxESI-MS1 adducts for d5-PC 17:0/22:4(*n*–6,9,12,15).

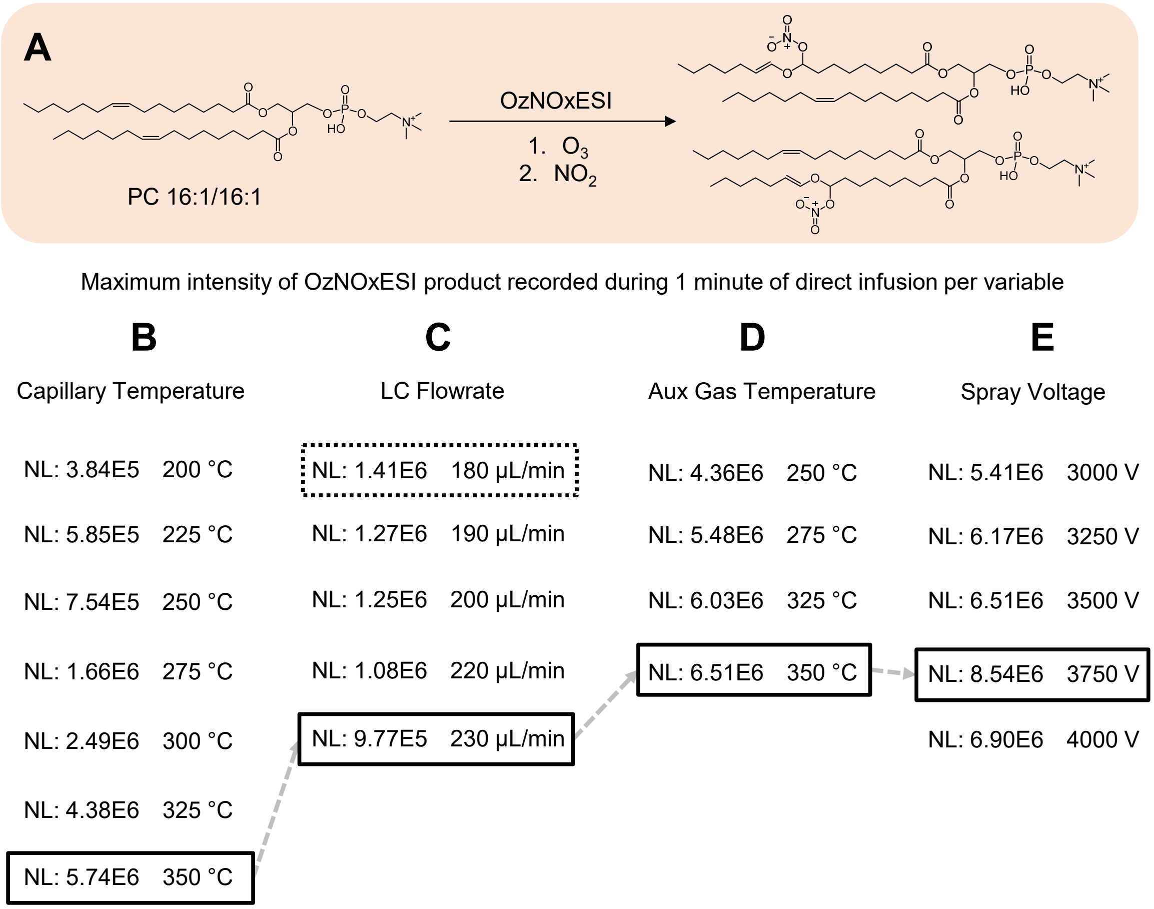

Figure S4. Stepwise optimization of HESI-II OzNOxESI by eluate infusion of PC standard.
(A) OzNOxESI reaction of PC 16:1(*n*–7)/16:1(*n*–7). The standard was introduced to column-less LC eluate via a syringe pump and PEEK T-junction just before the HESI-II source. The combined flowrate of the syringe pump and LC eluate was 230 µL/min. This imitates a continuously eluting LC-MS peak and allowed optimization under ionization plume conditions analogous to the planned lipidomics methodology, discussed further in the main text.
(B) The effect of capillary temperature on the MS detector intensity of the OzNOxESI product. Capillary temperature directly affects the sheath gas. The capillary temperature had the largest effect on OzNOxESI product yield, increasing by an order of magnitude from 200 °C to 350 °C. This indicates that the rate-limiting step in the OzNOxESI ion chemistry may be the *in situ* generation of nitric oxide (NO), which requires high temperature. Further temperature increase was not tested due to the concerns of the possible in-source fragmentation of analytes.
(C) The effect of total flowrate on the detector intensity of the OzNOxESI product. While keeping the syringe pump flowrate constant, the LC flowrate was reduced to decrease the total flowrate without altering the number of PC molecules entering the source. While lower flowrate did increase the intensity of the OzNOxESI product, we determined this increase did not justify adjusting the planned lipidomics gradient and methodology.
(D) The effect of auxiliary gas temperature on the detector intensity of the OzNOxESI product. The auxiliary gas provided to the source is just nitrogen in this work, and so it was expected that increasing this temperature would have less effect than the sheath gas. Still, increasing the auxiliary gas temperature did increase the product yield.
(E) The effect of spray voltage on the detector intensity of the OzNOxESI product. Increasing voltage increased OzNOxESI product intensity up to a point but then begins to introduce in-source fragmentation.

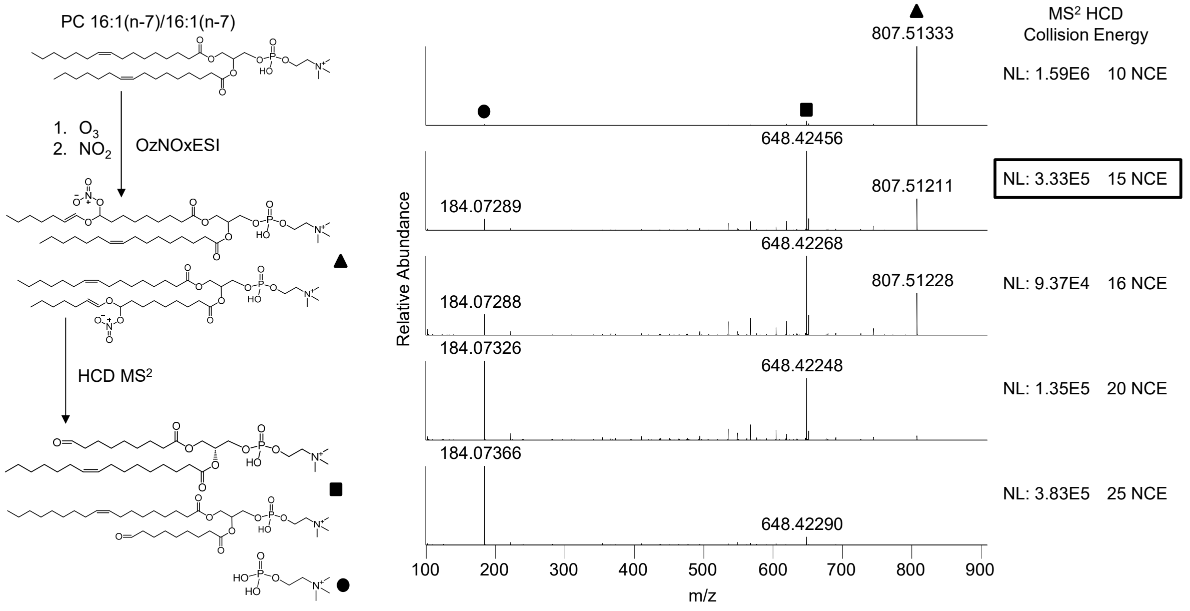

Figure S5. HCD collision energy optimization for LC-(+)-OzNOxESI-MS^2 analysis of PC lipids. The same eluate infusion approach described in Fig. S3 was used for optimizing HCD collision energies. Here, one product ion (*m/z* 648.42456) was observed corresponding to the C=C positions with intensity maximized at 15 NCE.

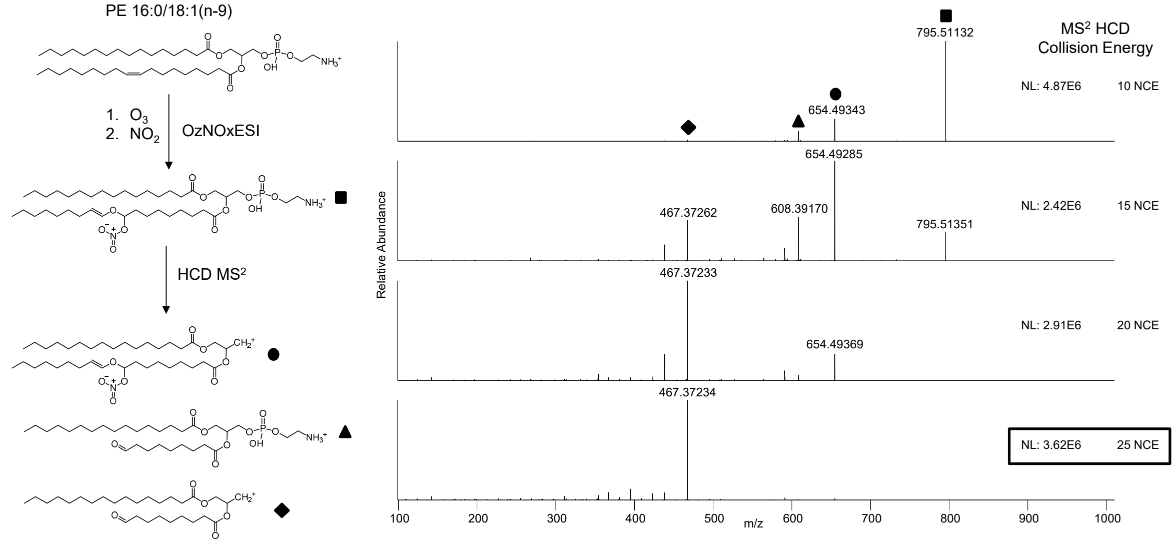

Figure S6. HCD collision energy optimization for LC-(+)-OzNOxESI-MS^2 analysis of PE lipids. Two product ions (*m/z* 608.39170 and 467.37262) were observed corresponding to the C=C position with highest intensity of either ion observed at 25 NCE.

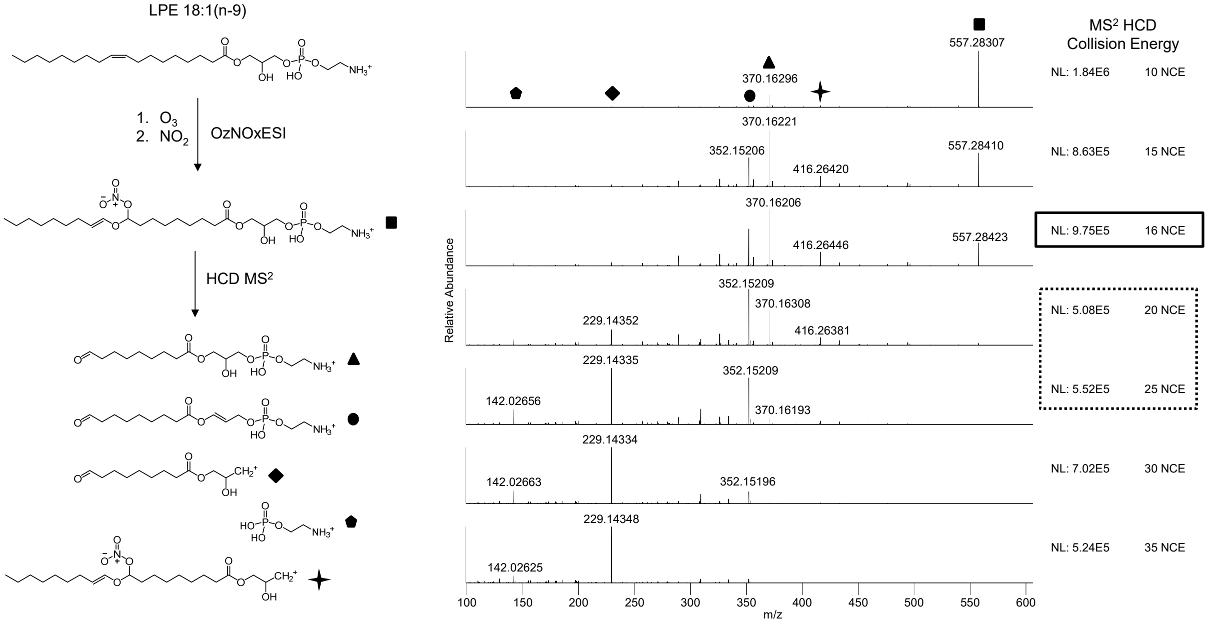

Figure S7. HCD collision energy optimization for LC-(+)-OzNOxESI-MS^2 analysis of LPE lipids. Three product ions were observed corresponding to the C=C position with highest intensity of any ion observed at 16 NCE. To promote a spectrum with greater diversity of diagnostic product ions at lower maximum intensity, the 20 – 25 NCE range may be preferred.

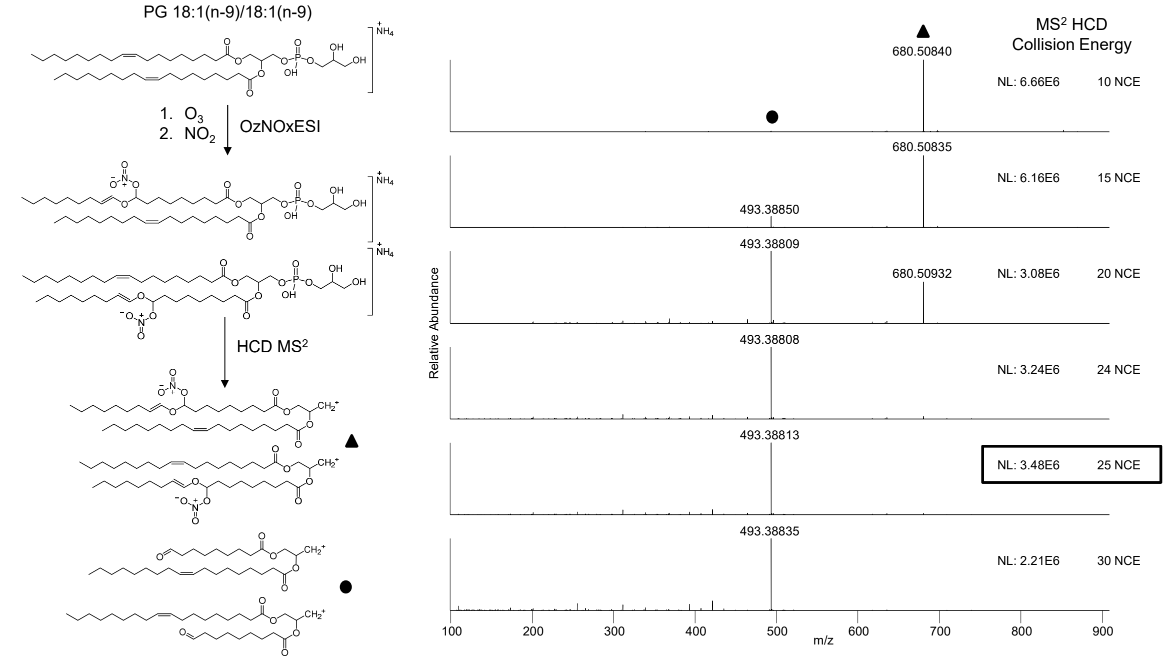

Figure S8. HCD collision energy optimization for LC-(+)-OzNOxESI-MS^2 analysis of PG lipids. One product ion was observed corresponding to the C=C positions with maximum intensity at 25 NCE.

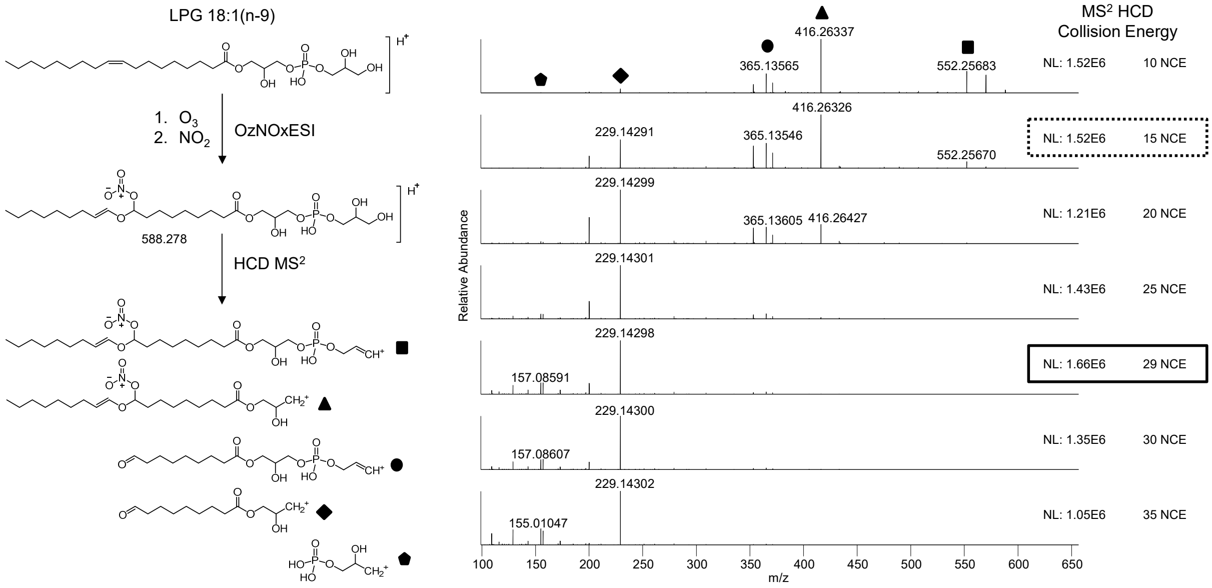

Figure S9. HCD collision energy optimization for LC-(+)-OzNOxESI-MS^2 analysis of LPG lipids. Two product ions were observed corresponding to the C=C position with highest intensity of either ion observed at 29 NCE. To promote a spectrum with greater diversity of diagnostic product ions at lower maximum intensity, 15 NCE may be preferred.

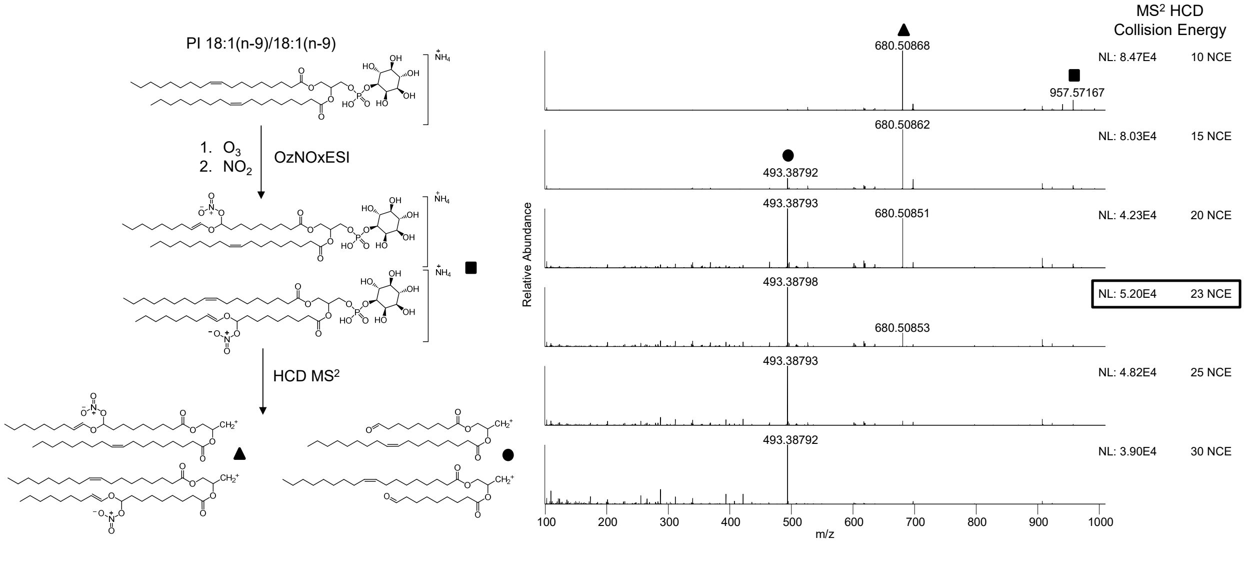

Figure S10. HCD collision energy optimization for LC-(+)-OzNOxESI-MS^2 analysis of PI lipids. One product ion was observed corresponding to the C=C positions with highest intensity observed at 23 NCE.

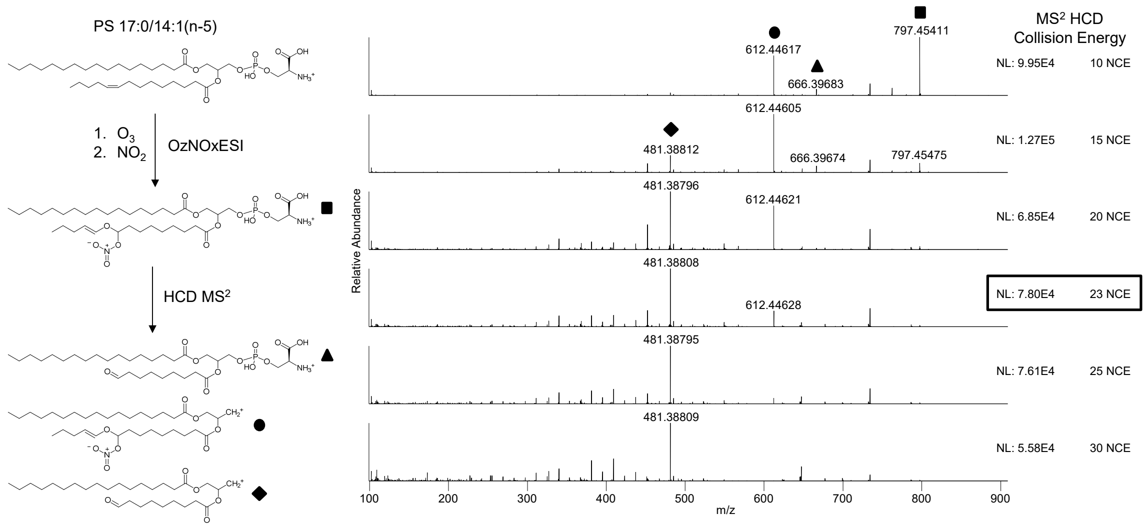

Figure S11. HCD collision energy optimization for LC-(+)-OzNOxESI-MS² analysis of PS lipids. Two product ions were observed corresponding to the C=C position with highest intensity of either ion observed at 23 NCE.

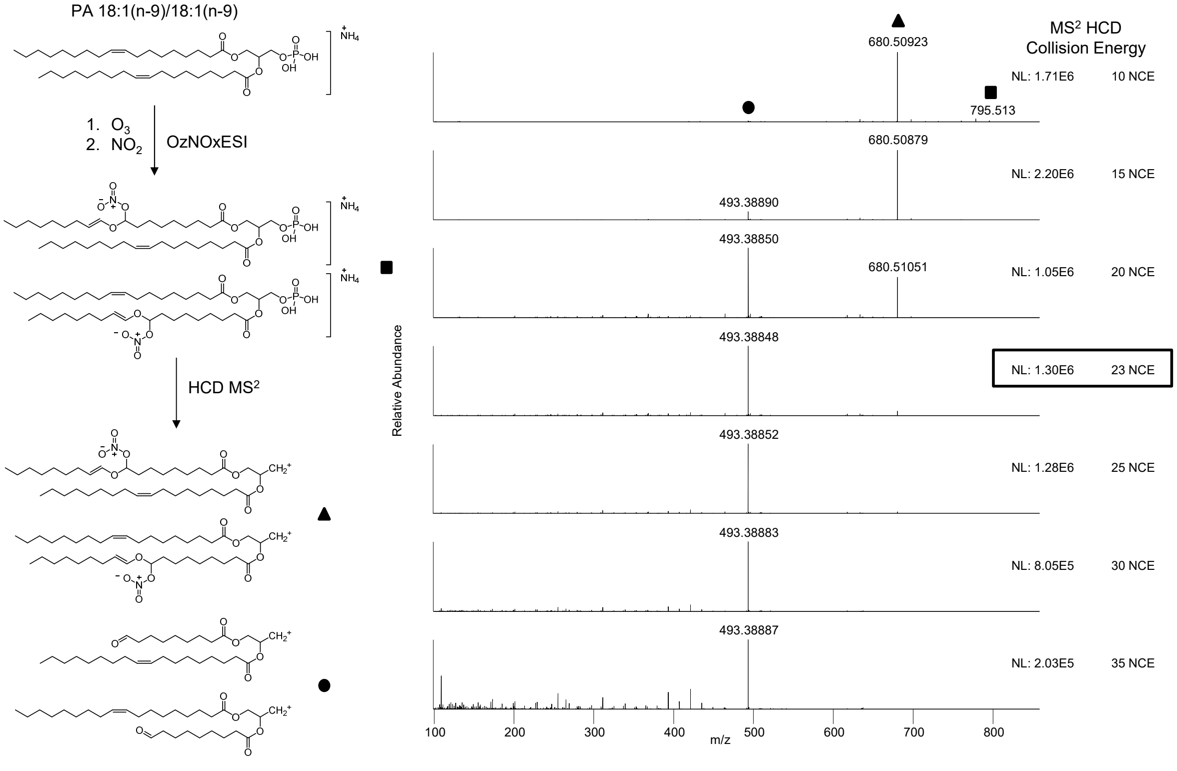

Figure S12. HCD collision energy optimization for LC-(+)-OzNOxESI-MS² analysis of PA lipids. One product ion was observed corresponding to the C=C positions with highest intensity observed at 23 NCE.

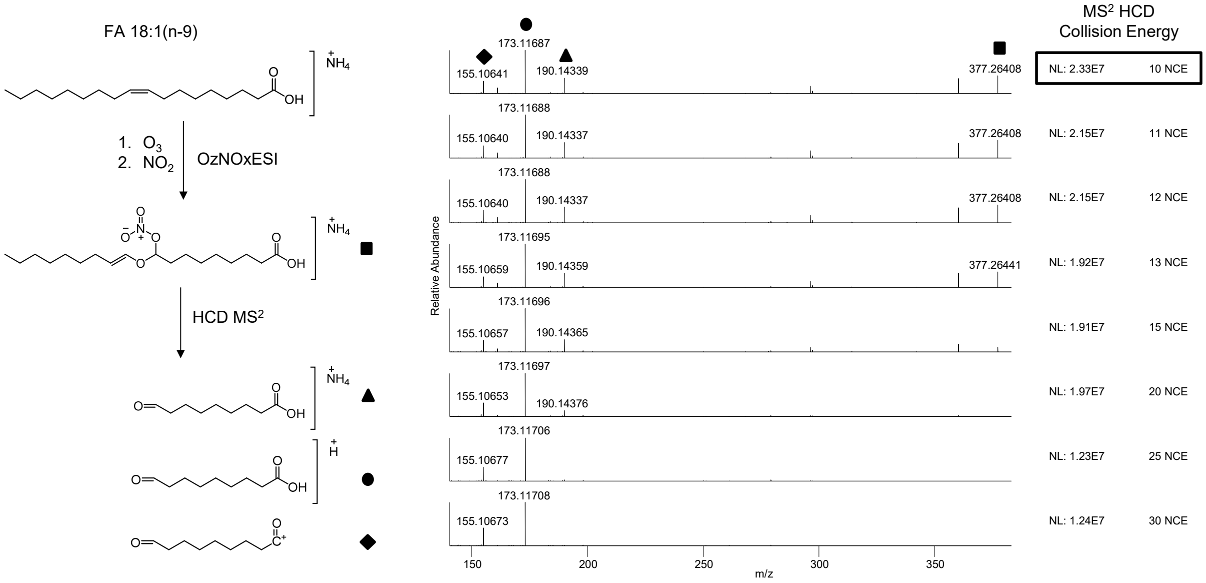

Figure S13. HCD collision energy optimization for LC-(+)-OzNOxESI-MS^2 analysis of fatty acids. Three product ions were observed corresponding to the C=C position with highest intensity of any ion observed at 10 NCE, which is the lower limit for collision energy on the Q Exactive HF instrument.

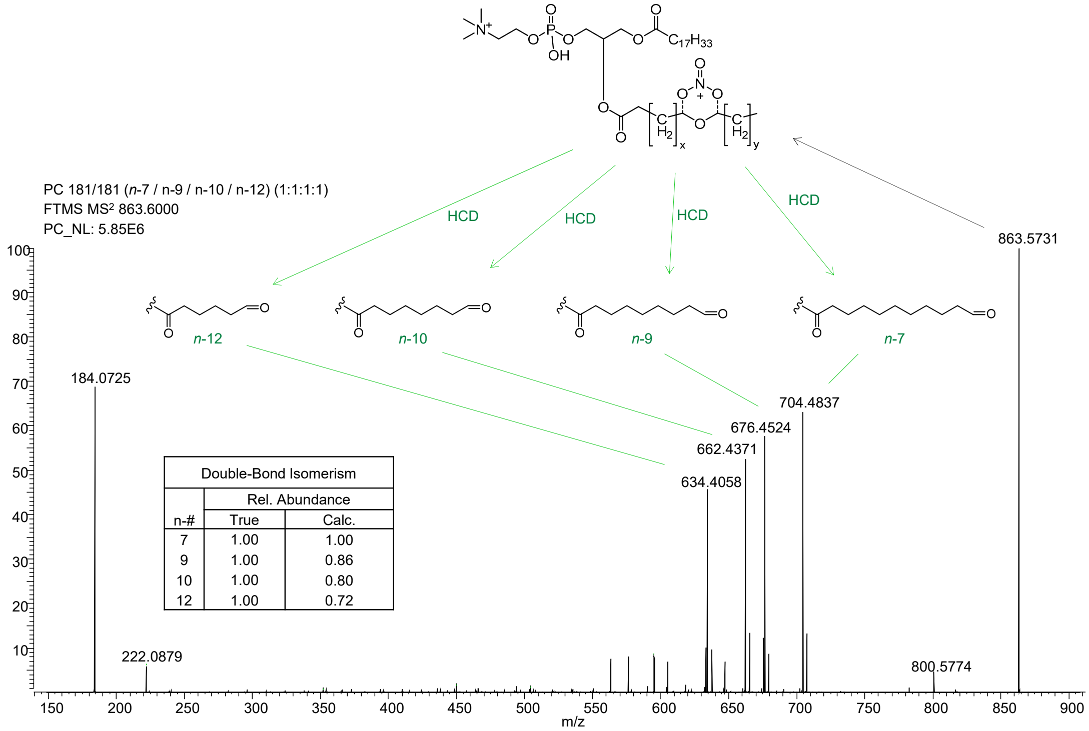

Figure S14. OzNOxESI-MS^2 acquisition of an equimolar mixture of the *n*-7, *n*-9, *n*-10, and *n*-12 isomers of PC 18:1/18:1. Analysis showed there is a slight difference in the abundances of OzNOx HCD-MS^2 products related to each isomer.

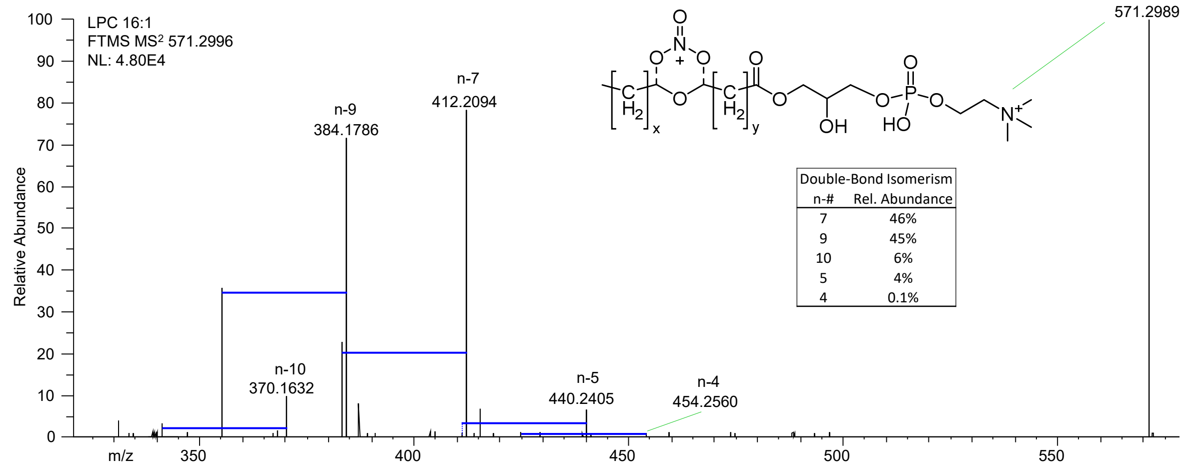

Figure S15. LC-OzNOx-MS^2 of LPC 16:1 from human plasma. The spectrum reveals a mixture of C=C regioisomers, predominantly LPC 16:1(*n*-7) and LPC 16:1(*n*-9).

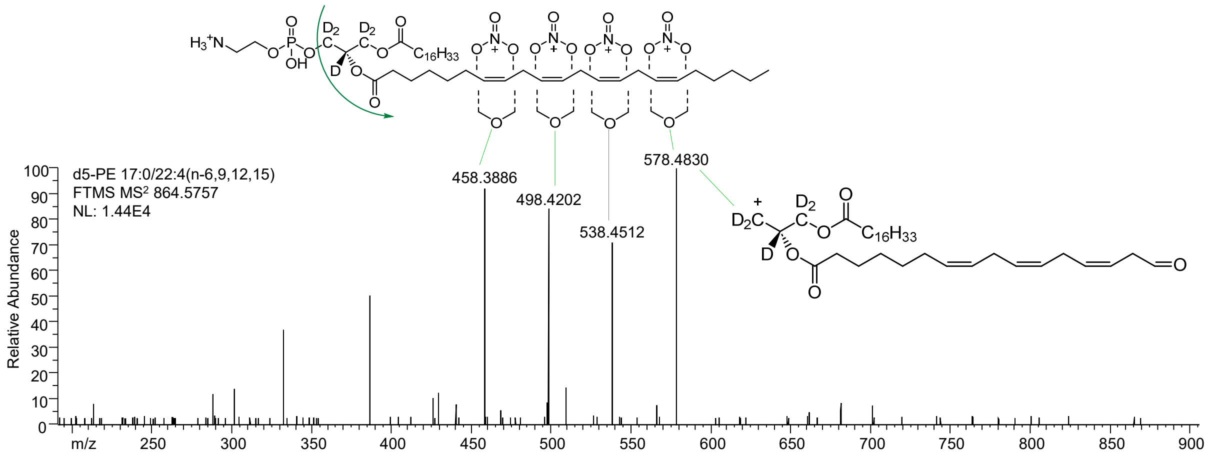

Figure S16. LC-OzNOxESI-MS^2 of internal standard d_5-PE 17:0/22:4(*n*-6,9,12,15). For each of the 4 C=C, there is a product ion analogous to the corresponding ozonolysis aldehyde, differing only by the loss of the head group (green arrow).

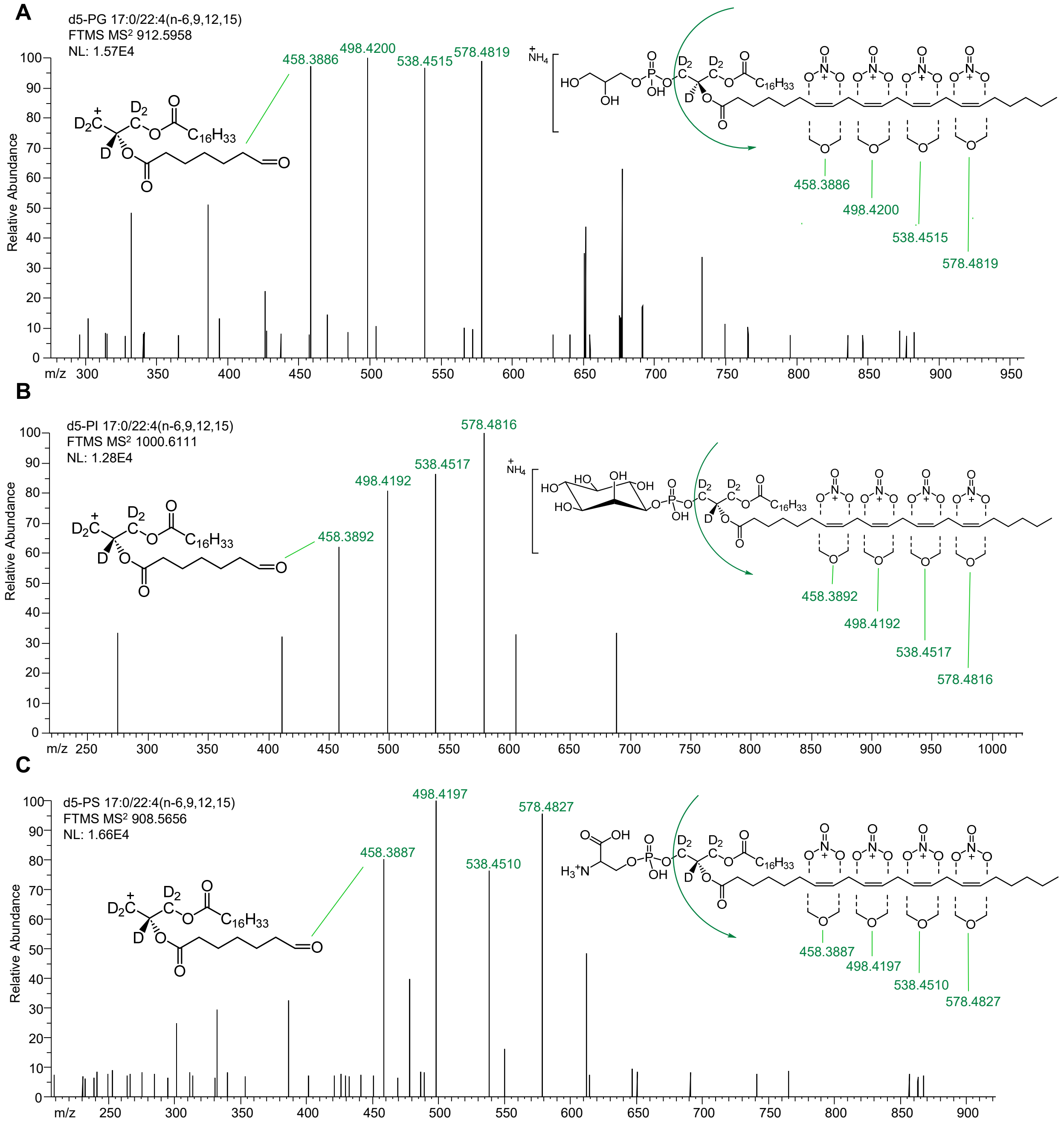

Figure S17. LC-OzNOxESI-MS^2 acquisition of representative PG, PI, and PS species. (A) OzNOx HCD-MS^2 of internal standard d5-PG 17:0/22:4(*n*–6,9,12,15). For each of the four C=C, there is a product ion analogous to the corresponding OzESI-MS^1 aldehyde, differing only by the loss of the head group (green arrow). (B) OzNOx HCD-MS^2 of internal standard d5-PI 17:0/22:4(*n*–6,9,12,15). The same fragmentation pattern is observed as in (A). (C) OzNOx HCD-MS^2 of internal standard d5-PS 17:0/22:4(*n*–6,9,12,15). The same fragmentation pattern is observed as in (A) and (B).

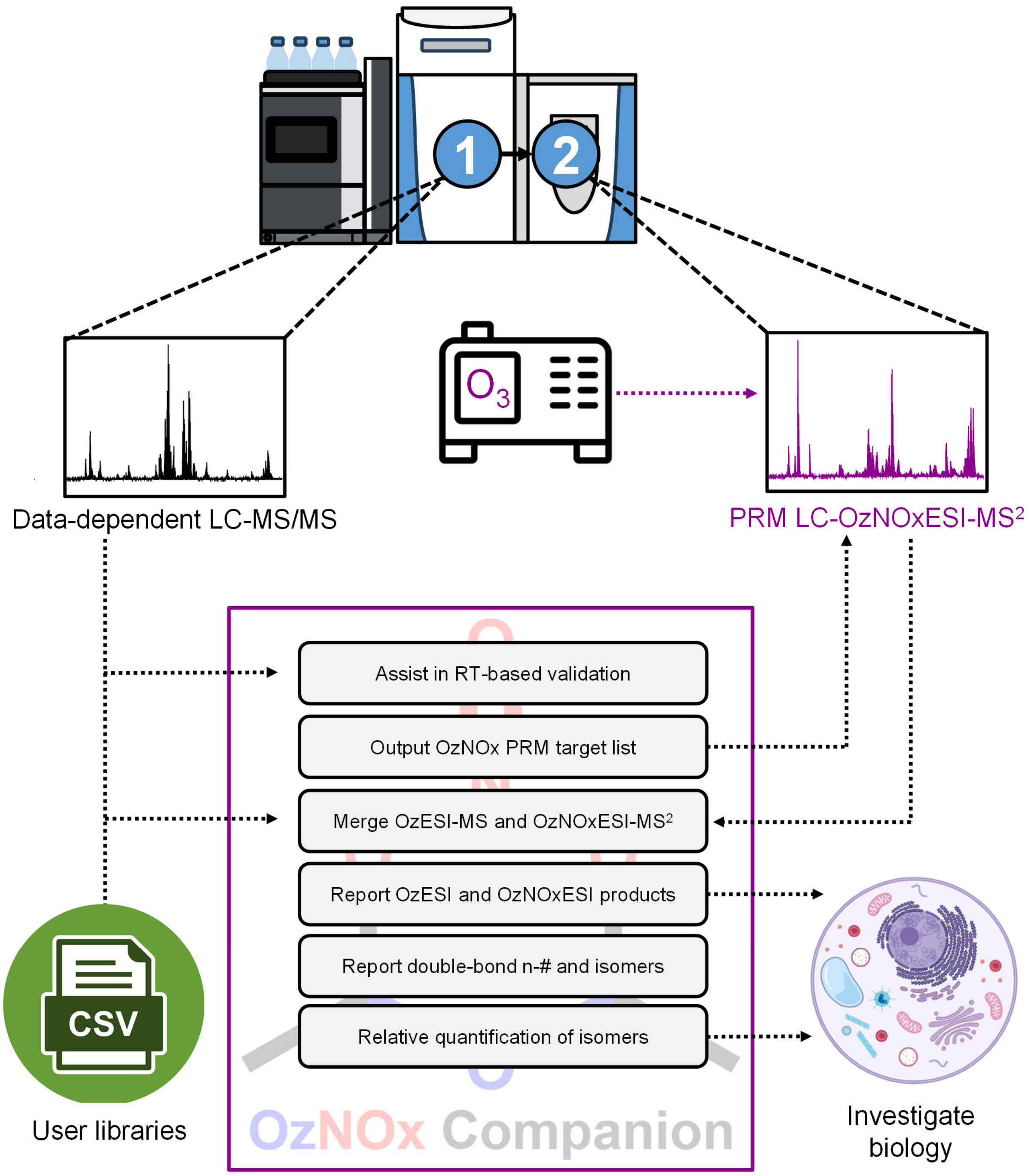

Figure S18. LC-OzNOxESI-MS/MS workflow with and functionality of OzNOx Companion. OzNOx Companion is a prototype software solution utilized in this study, designed to assist in each step of the LC-OzNOxESI-MS^2 workflow. OzNOx Companion contains eight scripts, each performing an essential step that is expedited by semi-automation. Editable user libraries and parameter codex are advantageous for allowing non-coder modification while refining instrument methodology. The program allows for multiple sample groups and alignment of replicate samples belonging to the same sample group. In the first scripts, the program assists in verification of initial annotations by RT-based inspection. Next, PRM target lists for approved annotations are provided in .csv format. Both OzESI-MS^1 and OzNOxESI-MS^2 products can be searched and reported from resulting data. Finally, C=C regioisomers and their relative quantification are calculated and reported.

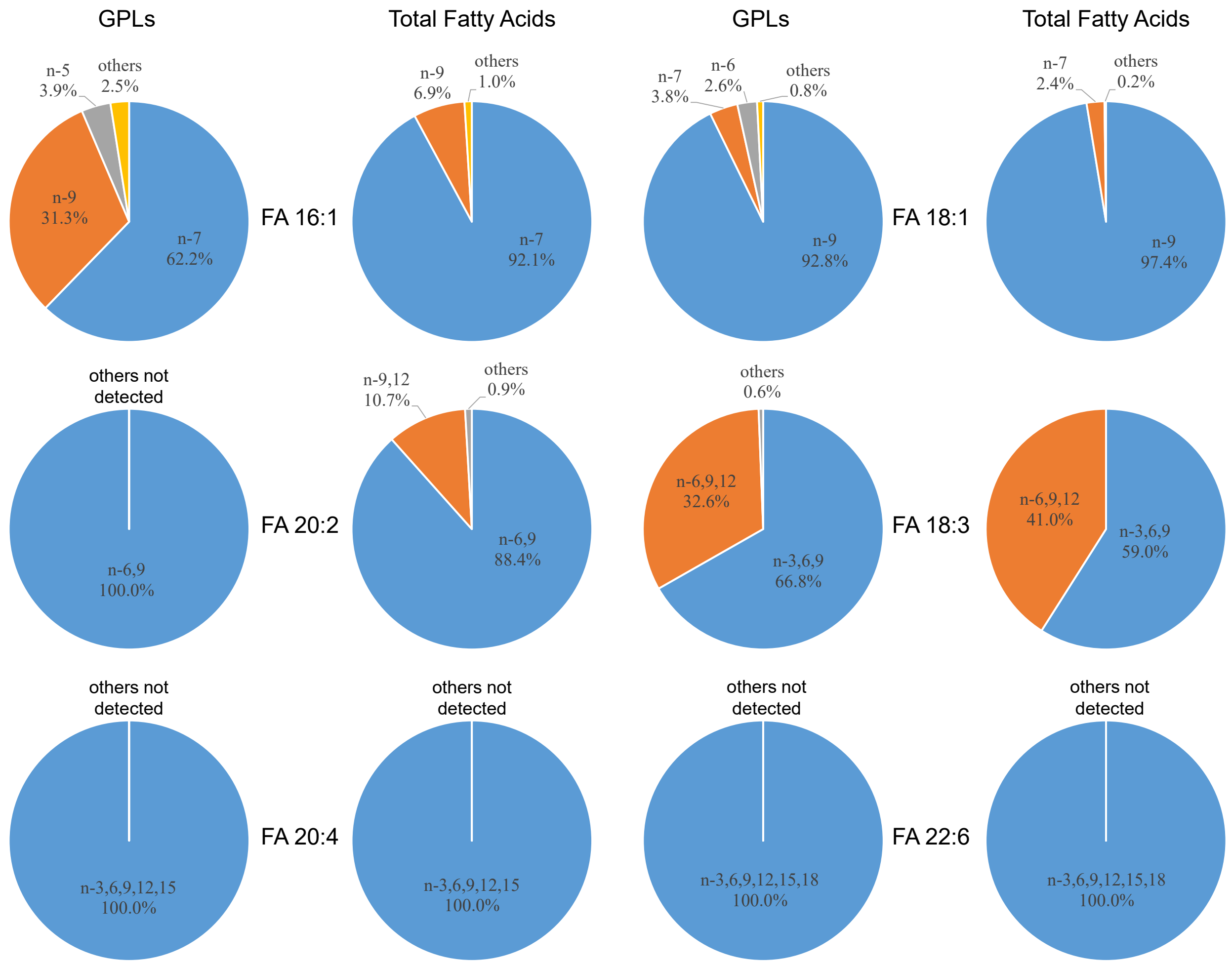

Figure S19. Distribution of C=C regioisomers from representative fatty acids in human plasma. LC-OzNOxESI-MS^2 was used in conjunction with OzNOx Companion to perform relative quantification of C=C regioisomers in GPL-bound fatty acyls and in fatty acids hydrolyzed from total lipid extract. For fatty acids FA 16:1, FA 18:1, FA 20:2, FA 18:3, FA 20:4, and FA 22:6, the C=C regioisomerism of GPL-bound fatty acyls (left) and total fatty acids (right) are presented for comparison and show consistent similarity. GPL fatty acyls are expected to have similar but not necessarily identical regioisomerism to the total fatty acids of the same sample.
